# Rational Nanomedicine Design Enhances Clinically Physical Treatment‐Inspired or Combined Immunotherapy

**DOI:** 10.1002/advs.202203921

**Published:** 2022-08-24

**Authors:** Qiaoqiao Liu, Wei Zhang, Rong Jiao, Zheng Lv, Xia Lin, Yunping Xiao, Kun Zhang

**Affiliations:** ^1^ Department of Radiology Liuzhou People's Hospital Affiliated to Guangxi Medical University No. 8 Wenchang Road Liuzhou 545006 P. R. China; ^2^ Central Laboratory Shanghai Tenth People's Hospital Tongji University School of Medicine Shanghai 200072 P. R. China; ^3^ National Center for International Research of Bio‐targeting Theranostics Guangxi Key Laboratory of Bio‐targeting Theranostics Guangxi Medical University No. 22 Shuangyong Road 22 Nanning 530021 P. R. China

**Keywords:** clinical physical treatment, immunity activation, nanomedicine, thermal ablation, ultrasound treatment

## Abstract

Independent of tumor type and non‐invasive or minimally‐invasive feature, current physical treatments including ultrasound therapy, microwave ablation (MWA), and radiofrequency ablation (RFA) are widely used as the local treatment methods in clinics for directly killing tumors and activating systematic immune responses. However, the activated immune responses are inadequate and incompetent for tumor recession, and the incomplete thermal ablation even aggravates the immunosuppressive tumor microenvironment (ITM), resulting in the intractable tumor recurrence and metastasis. Intriguingly, nanomedicine provides a powerful platform as they can elevate energy utilization efficiency and augment oncolytic effects for mitigating ITM and potentiating the systematic immune responses. Especially after combining with clinical immunotherapy, the anti‐tumor killing effect by activating or enhancing the human anti‐tumor immune system is reached, enabling the effective prevention against tumor recurrence and metastasis. This review systematically introduces the cutting‐edge progress and direction of nanobiotechnologies and their corresponding nanomaterials. Moreover, the enhanced physical treatment efficiency against tumor progression, relapse, and metastasis via activating or potentiating the autologous immunity or combining with exogenous immunotherapeutic agents is exemplified, and their rationales are analyzed. This review offers general guidance or directions to enhance clinical physical treatment from the perspectives of immunity activation or magnification.

## Graphical abstract

Nanomedicine‐enhanced physical therapy (e.g., SDT, HIFU, RFA, and MWA) can stimulate and induce robust anti‐tumor immune responses in vivo to a certain extent. Especially when combining with immunotherapy drugs, it shows a considerably‐enhanced anti‐tumor effect which can not only treat primary tumors but also effectively repress metastatic tumors, thus holding great potential in tumor prevention.

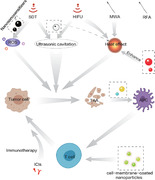

## 1 Introduction

Cancer is still one of the major human health burdens and greatly endangers human life.^[^ ^1^, ^2^ ^]^ Differing from conventional treatment methods^[^ ^3^, ^4^, ^5^ ^]^ that suffer from a series of side effects,^[^ ^6^, ^7^ ^]^ physical treatments featuring high efficiency, high specificity, and low side effects are urgently expected and acquire significant breakthroughs in repressing tumor progression and reducing the recurrence and metastasis rates.^[^ ^8^ ^]^ Typically, some clinically‐common physical treatment approaches such as ultrasound (US) treatment including sonodynamic therapy (SDT) and high‐intensity focused ultrasound (HIFU) ablation, microwave ablation (MWA), and radiofrequency ablation (RFA) featuring non‐invasive and minimally‐invasive penetration are receiving increasing attention. These means are anticipated to address the above side effects that conventional treatment methods encountered and realize the objective expectations because they, as the general method, can exert the potent anti‐tumor actions without dependence on the type, site, and malignancy degree of cancers.

**Figure 1:**
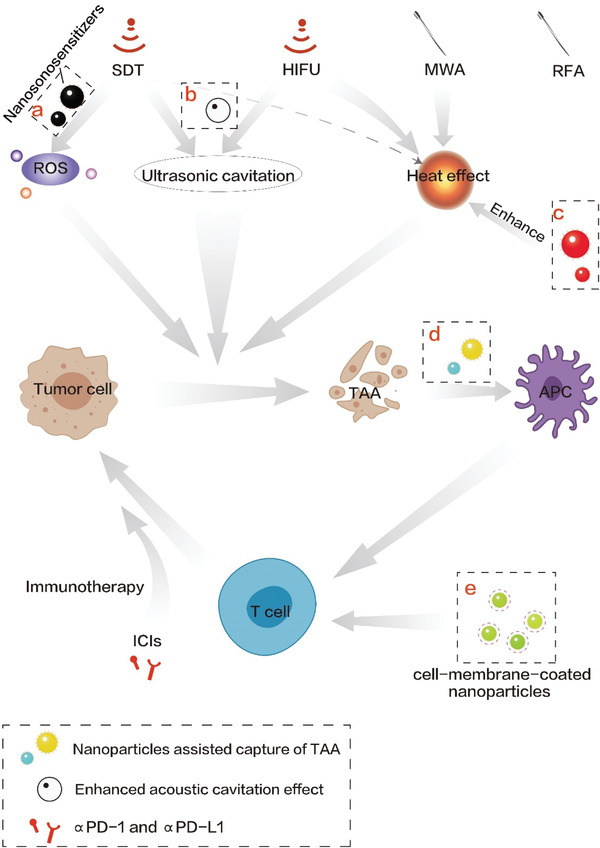
a) Nanosonosensitizers promoted the production of ROS. b) Acoustic cavitation effect enhanced by nanoparticle‐based cavitation nuclei. c) Nanoparticles enhanced the efficiency of thermal ablation. d) Nanoparticles captured tumor‐associated antigens (TAA) and presented them to antigen presenting cells (APCs). e) Cell‐membrane‐coated nanoparticles directly activated T cells.

Nevertheless, the local operation manner dictates the limited immune responses of these physical treatment methods. Regarding this, a surge of combined therapy with immunotherapy or nanomedicine to boost the immune responses, is accessible.^[^ ^9^, ^10^, ^11^, ^12^, ^13^ ^]^ In particular, various nanomedicines and nanobiotechnologies such as photodynamic therapy (PDT),^[^ ^14^, ^15^, ^16^, ^17^ ^]^ photothermal ablation (PTA),^[^ ^18^, ^19^ ^]^ radiotherapy,^[^ ^20^ ^]^ chemodynamic therapy (CDT) or nanocatalytic medicine,^[^ ^21^, ^22^ ^]^ have been documented to activate immune responses and potentiate immunotherapy after combining them (e.g., immune checkpoint blockade). Great efforts and significant advances have been made in these combined therapies to resist tumor recurrence or metastasis via directly elevating energy utilization efficiency, remodeling immunosuppressive tumor microenvironment (ITM) and increasing antigen exposure, and presentation and promotion of immune cell infiltration.^[^ ^23^, ^24^, ^25^, ^26^ ^]^ These inspiring cases provide experiences and basis to the combined therapies of clinically physical treatment methods with immunotherapy under the assistance of nanomedicine or nanobiotechnologies. Nanomedicine alone could induce some biophysical activities of these clinically physical treatment methods via inducing reactive oxygen species (ROS) production, and enhancing inertial cavitation and/or elevating heat effect, which are harnessed to promote the birth, exposure, and presentation of antigens and activate the autologous immune responses to exert anti‐tumor immunotherapy without adding exogenous immunotherapeutic agents,^[^ ^27^, ^28^, ^29^ ^]^ as evidenced in **Figure** **1**. More significantly, other imaging unit integration confers them with imaging‐guided function, which is preferable for treatment precision elevation and treatment progress monitoring.^[^ ^30^, ^31^, ^32^ ^]^ Previous reviews focused on the nanomedicine or nanobiotechnology‐enhanced energy utilization of each physical treatment and discussed the rationales of nanomedicine, while few reviews systematically highlighted the autologously activated immune responses or combined with exogeneous immunotherapeutic agents to enhance the anti‐tumor outcomes of the above clinically physical treatment methods.

In this review, we briefly introduced some signal pathways related to tumor immunity, with emphasis on the progress of nanobiotechnologies. The corresponding nanomaterials that could furnish reliable tools to enhance physical treatment efficiency against tumor progression, relapse, and metastasis via activating or potentiating the autologous immunity or combining with exogenous immunotherapeutic agents was underlined. Herein, we first offered an outlined glimpse at the category and principle of each physical treatment means, based on which the cutting‐edge progress and direction associated with efficiency elevation and immune activation were emphasized. Subsequently, we expatiated the roles of rational nanomedicine design in the combined therapy of common physical treatments (ultrasound, MWA, RFA, and RFA) with immunotherapy with some typical paradigms and analyzed their rationales. More significantly, we highlighted the activation and magnification of systematic immune responses using the nanomaterials when referring to enhancing the local physical treatment efficiency. Eventually, we pointed out the unresolved concerns, provided the potential solution, and discussed the challenge and direction of physical treatment and future fundamental research so as to guide new development of nanomedicine and nanobiotechnology in clinical physical treatments.

## 2 Classic Immune Signal Pathway

The innate immune system can recognize pathogens and identify dead and abnormal cells in the body, thus initiating a protective response. This recognition relies on pattern recognition receptors (PRRs) that can sense pathogen‐related molecular patterns (PAMP) and damage‐related molecular patterns (DAMP). Among them, membrane‐bound PRRs contain ToII‐like receptors (TLRs) and retinoic acid‐induced gene I (RIG‐I) like receptors, while cytoplasmic PRRS contain NOD‐like receptors (NLRs). NLR can be divided into NLRP and NLRC. In addition, cGAS‐STING can recognize a wide range of double‐stranded DNA (dsDNA) and participate in immune activation pathways.

### 2.1 TLRs

TLRs are one of the PRRs and are accepted to be the important in vivo anti‐pathogen or endogenous release molecules, which are made up of extracellular leucine‐rich repeat sequence (LRR) domains, while intracellular Toll/IL‐1 receptors (TIR) domains constitute the type I transmembrane receptor family.^[^ ^33^, ^34^ ^]^ Therein, LRR interacts with DAMP, which allows TIR domain to bind to cytoplasmic proteins, causing the activation of downstream networks, such as C‐Jun N‐terminal kinase, NF‐kB, and p38 MAP kinase pathway.^[^ ^35^ ^]^ Agonists of TLR receptors have been discovered and applied to tumor immunotherapy. As immunomodulators, TLR agonists can stimulate natural killer cells (NK) and cytotoxic lymphocytes, induce dendritic cells (DCs) maturation to activate innate immunity, and cause long‐term adaptive immunity, which can also enhance the sensitivity of tumor cells to other treatments such as chemotherapy, radiation therapy, and immunotherapy.^[^ ^36^ ^]^

### 2.2 RIG‐I

The retinoic acid‐inducible gene I (RIG‐I)‐like receptor (RLRs) is another pattern‐recognition receptor that recognizes molecules of double‐stranded RNA (dsRNAs) and ssRNA virus pathogens, constituting a defense line against RNA viruses. Short dsRNA with the 5'‐triphosphate moiety is the specific ligand of RIG‐I, and when binding with RNA, RIG‐I activates interferon regulator factor 3 (IRF3), mitose‐activated protein kinases, and NF‐kB pathway through mitochondrial antiviral signaling proteins (MAVS). In addition to playing an important role in the induction of antiviral cytokines, RIG‐I causes tumor cell death through RIG‐I–independent and IFN‐I–primed mechanisms.^[^ ^37^, ^38^ ^]^

### 2.3 NLRP3 Inflammasome

NLRP3 inflammasome is an important component of the response system to microbial infection and cellular damages, and it is a multimeric cytoplasmic protein complex produced in response to pathogen‐associated molecular patterns (PAMP) and damage‐associated molecular patterns (DAMP).^[^ ^39^, ^40^ ^]^ NLRP3 can activate cysteine protease caspase‐1 to promote IL‐1*β* and IL‐18 secretions as well as inflammatory cell death. Tumor‐associated inflammation is caused by inflammasome and by a variety of immune cells such as macrophages, dendritic cells, natural killer cells (NK), neutrophils, and T and B lymphocytes.^[^ ^41^ ^]^ In the course of fighting against tumors, the impact of NLRP3 inflammasome on tumor immunity has a double‐edged role, which is decided by tumor type. In brief, it plays an inflammatory protective role in colorectal cancer, while it favors invasive growth of breast cancer, gastric cancer, lung cancer, and so on.^[^ ^42^ ^]^

### 2.4 cGAS‐STING

Cyclic GMP‐AMP synthase (cGAS)–interferon gene stimulator (STING) pathway is an important DNA sensing mechanism in innate immunity and virus defense, which can be activated by dsNDA. In this pathway, cGAS interacts with dsDNA to activate its catalytic activity, leading to the production of the second messenger molecule, that is, 2 “3” cyclic GMP‐AMP (cGAMP). Then, cGAMP activates STING in the endoplasmic reticulum to recruit TANK binding kinase 1 (TBK1) protein and phosphorylates, activates interference regulator factor 3 (IRF3), and induces type 1 interferon after they enter nuclei to activate innate immunity. cGAS can recognize a wide range of foreign and self‐originated DNA, and its role has been extended to cancer.^[^ ^43^, ^44^ ^]^

## 3 Clinical Physiotherapy‐Activated or Combined Immunotherapy

Currently, the prevalent physical treatment methods include ultrasound treatment, MWA treatment, and RFA treatment. According to their treatment principles, different nanotechnology and nanomaterials were designed to elevate the energy transformation efficiency for acquiring high physical treatment outcomes. High physical treatment efficiency can activate systematic immune responses including more antigen exposures and presentations, ITM mitigation, and effector T infiltration, to favor autologous immunotherapy and combined immunotherapy against tumor progression, relapse, and metastasis. As a result, nanotechnology or nanomaterials‐enhanced physical treatment is the premise of autologously‐activated or combined immunotherapy.

### 3.1 Ultrasound Treatment

Ultrasound is a mechanical wave, which can be used in ultrasonic diagnosis and ultrasound treatment.^[^ ^45^, ^46^, ^47^ ^]^ In terms of treatment, two types of US treatment methods were classified; one is SDT and another is HIFU ablation, both of which are non‐invasive methods with deep tissue penetration, high efficiency, low side effects, and low cost.^[^ ^48^ ^]^ In terms of clinical application of SDT, a breast cancer‐bearing patient aged 55 years received a combined SDT including Gc protein‐derived macrophage‐activating factor (GcMAF) and hormone therapy in a medical record report. After the treatment, the symptoms were improved significantly, for example, lesion disappearance and tumor marker decrease, with which slight side effects caused by hormones were accompanied.^[^ ^49^ ^]^ In another pathological report, a patient aged 77 years with non‐small cell lung cancer received GcMAF and oral colostrum MAF‐based immunotherapy that were combined with SDT, TTF, and Ozone therapy. The level of tumor marker (i.e., neuron‐specific enolase [NSE]) was decreased to within the normal range, and the tumor volume did not increase within 15 months.^[^ ^50^ ^]^

SDT depends on sonosensitizers, where low‐intensity ultrasound irradiates sonosensitizers to stimulate immobilized electron transfer to adjacent free oxygen‐contained species and produce ROS to kill tumor.^[^ ^51^, ^52^, ^53^, ^54^ ^]^ HIFU depends on the hyperpyrexia, where the mechanical energy of ultrasound waves is switched into heat to ablate tumor.^[^ ^55^ ^]^ In the field of acoustics, ultrasound cavitation is usually accompanied, which is available for benefiting SDT and HIFU.^[^ ^56^, ^57^ ^]^ Therefore, cavitation is identified as the origin of ultrasound treatment as it often induces some biophysical effects to enable SDT or HIFU potentiation.^[^ ^28^, ^55^ ^]^ Generally, cavitation is classified into inertial cavitation and stable cavitation.^[^ ^55^, ^58^ ^]^ As cavitation proceeds, energy is released to give birth to shock waves, microjets, high temperature, high pressure, and so on,^[^ ^59^, ^60^ ^]^ and these biophysical effects could be used enhance HIFU temperature and sonocatalytic ROS production for killing tumor cells.

#### 3.1.1 Design Rationales of Nanobiotechnologies‐Augmented SDT

**Figure 2:**
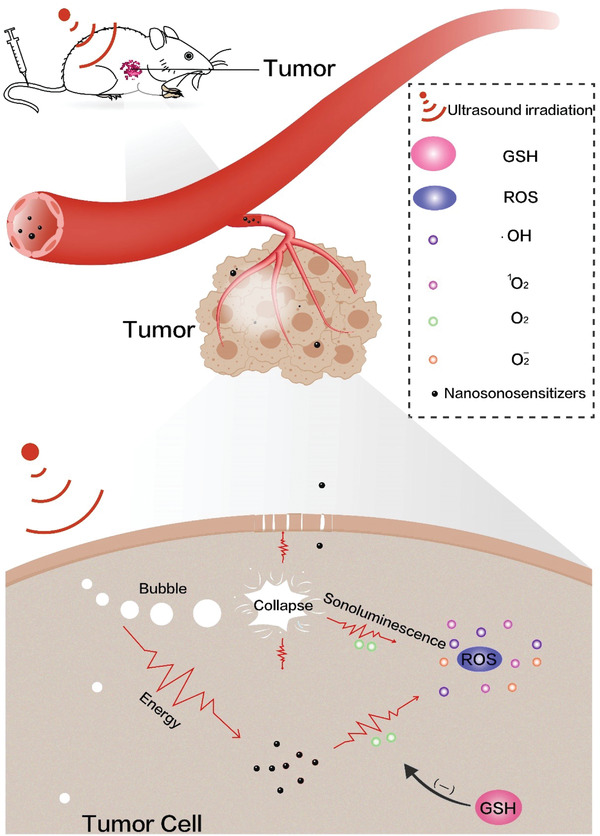
Therapeutic mechanism of SDT under the action of sonosensitizers.

With the comprehensive and deep understandings of SDT, SDT principle was progressively figured out, as outlined in **Figure** **2**. In detail, several steps were generalized, sonoluminescence process,^[^ ^48^ ^]^ sonosensitizers‐mediated electron (e^–^)/hole–(h^+^) pairs separation, electron transfer to adjacent free oxygen‐contained molecules for producing ROS (e.g., ^1^O_2_, ·OH, etc.), and the last irreversible damages to the tumor cells and induced tumor cell necrosis or apoptosis.^[^ ^59^, ^61^, ^62^ ^]^ In SDT, sonosensitizers are indispensable to convert mechanical energy of ultrasound waves into ROS‐represented chemical energy. Generally, sonosensitizers cover organic nanosonosensitizers and inorganic nanosonosensitizers.^[^ ^8^ ^]^ In light of the fact that SDT are considered to derive from PDT,^[^ ^56^, ^63^ ^]^ most photosensitizers usually serve as sonosensitizers, which were loaded in nanocarriers to improve the bioavailability, pharmacokinetics, biosafety, and ultrasonic stability.^[^ ^64^ ^]^

**Figure 3:**
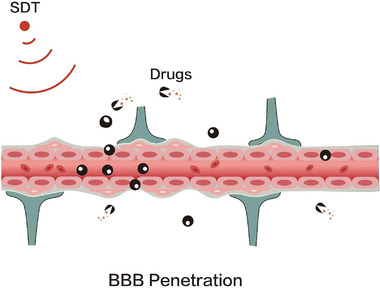
Schematic illustration of using SDT to destroy the blood–brain barrier (BBB) instantaneously and reversibly for greatly promoting the intracerebral delivery of drugs.

The level of sonocatalytic ROS production is identified as the source of SDT and also closely correlates with immunotherapy as ROS level dictates immunogenic cell death (ICD) and mitigates ITM to magnify autologous immunotherapy and combined immunotherapy.^[^ ^28^ ^]^ It determines that developing sonosensitizers featuring high catalytic activity is highly desirable and is regarded as one direction to elevate ROS level. As materials science and nanotechnology proceed, various pathways referring to design sonocatalytic active sites‐contained nanomaterials have been developed, wherein the electron (e^–^)–hole (h^+^) separation and re‐combination inhibition were first highlighted via various methods, for example, surface functionalization,^[^ ^56^, ^65^ ^]^ noble metal doping,^[^ ^66^ ^]^ and 2D nanosheets supporting.^[^ ^51^, ^64^, ^67^ ^]^ In addition, thermal effect was found to boost the sonocatalytic activity of SDT and benefit ROS production.^[^ ^68^ ^]^ In particular, the recent advances in the field of SDT reveal that the piezoelectric materials and nonstoichiometric metal‐based nanomaterials with rich inherent defects are sprouted and attract increasing attention as another appealing route for enhancing ROS production.^[^ ^69^, ^70^, ^71^, ^72^ ^]^ In detail, the rich defects in burgeoning nonstoichiometric metal‐based sonosensitizers allow the free and unpaired electrons to activate adsorbed oxygen‐contained species to generate ROS.^[^ ^73^, ^74^ ^]^ The piezoelectric materials will be deformed upon experiencing sound pressure and then the electron‐hole pairs are separated to induce ROS birth.^[^ ^72^, ^75^ ^]^ Impressively, the acoustic cavitation dose correlates with ROS generation because cavitation can split H_2_O and further stimulate the sonosensitizers to produce electron (e^–^)–hole (h^+^) pairs and then, ROS evolution.^[^ ^52^, ^76^, ^77^ ^]^ Regarding this point, exogenous cavitation nuclei are introduced to augment ROS level, which are also used to combine with drug delivery to further elevate the anti‐tumor outcome (**Figure** **3**).^[^ ^78^ ^]^

**Figure 4:**
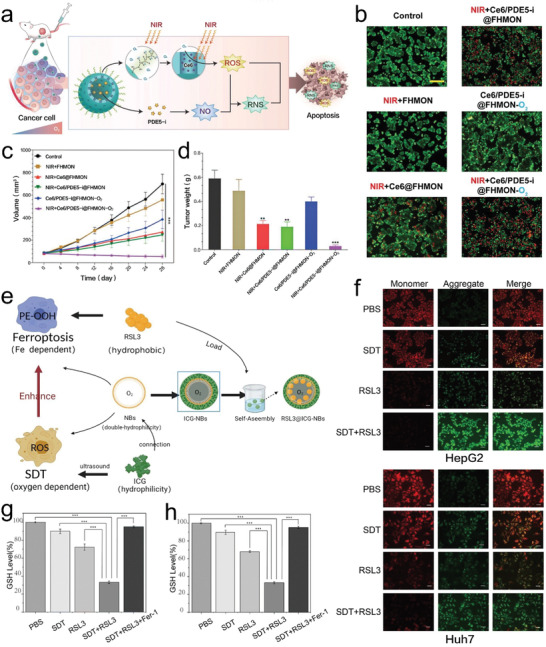
a) The dynamic N/O balance disruption for enhancing ROS‐based anti‐tumor via switching ROS into RNS with longer life‐time. b) Confocal laser scanning microscopy (CLSM) images of cell treated with different groups. c) Tumor volume changes in each treatment group during the experiment. d) Tumor weight at the end of the experiment. Reproduced under the terms of the Creative Commons CC‐BY license.^[^ ^80^ ^]^ Copyright 2021, The Authors. Published by Wiley‐VCH. e) Schematic of self‐assembled RSL3@O_2_‐ICG NBs for enhancing SDT and ferroptosis. f) CLSM images of JC‐1 monomers (green channel) and aggregates (red channel) in mitochondria of HepG2 and Huh7 cells in different experimental groups. g,h) GSH levels in HepG2 (g) and Huh7 (h) cells treated with different experimental groups. Reproduced with permission.^[87^ ^]^ Copyright 2022, Dove Press Ltd.

Besides rationally developing new sonosensitizers, tumor microenvironment (TME) also decides ROS production. It is well‐known that ROS production process requires oxygen.^[^ ^79^, ^80^ ^]^ Actually, solid tumor tissue is usually hypoxic due to the abnormal metabolism, which will greatly discount the production of ROS and then affect the therapeutic effect of SDT.^[^ ^70^, ^81^ ^]^ Therefore, hypoxia mitigation to supply adequate oxygen grasped the essence, and various O_2_‐release nanomaterials using microbubble, perfluorocarbon or hemoglobin‐mediated O_2_ delivery,^[^ ^25^, ^81^ ^]^ photo‐ or electron‐catalytical O_2_ production,^[^ ^76^ ^]^ Fenton or Fenton‐like O_2_ release,^[^ ^20^ ^]^ and so on as the design rationales, have been rationally constructed. The O_2_ release could not only mitigate hypoxic TME but also liberate the hypoxia‐induced resistance to ROS‐based anti‐tumor actions.^[^ ^20^, ^28^ ^]^ Besides hypoxia, redox balance is another TME component which can resist ROS accumulation via depleting ROS with reducing agents (e.g., glutathione [GSH]).^[^ ^16^, ^51^ ^]^ Regarding this, GSH depletion and GSH synthesis blockade are two means to reduce the intratumoral GSH level or disrupt GSH metabolism,^[^ ^16^, ^51^, ^82^ ^]^ break the redox balance, and favor abundant ROS accumulation in tumor.^[^ ^83^, ^84^ ^]^ Notably, nitrogen/oxygen balance disruption is also favorable for enhancing ROS‐based anti‐tumor outcome.^[^ ^80^, ^85^ ^]^ It is found that switching ROS into reactive nitrogen species (RNS) was undertaken to prolong the anti‐tumor action time as RNS have longer lifetime than ROS and can circumvent ROS annihilation before they can enter and destroy cell nuclei or mitochondria (**Figure** **4**a). They constructed a nano‐sized platform that could produce ROS and switch them into RNS, eventually receiving the most robust anti‐tumor effects against MCF‐7 cells in vitro (Figure 4b) and significantly inhibiting the growth of MCF‐7 tumors in vivo (Figure 4c,d).

Genetic‐engineering technology was also combined to modulate TME to enhance SDT. Pu et al. developed a metal–organic skeleton (MOF) integrated with acoustic‐controllable and ROS‐sensitive sonosensitizers using gene‐editing technology.^[^ ^86^ ^]^ The nanoparticles could convert ultrasonic energy into ^1^O_2_ and then induce the controllable release of CRISPR‐Cas9 system to initiate genome editing. The ROS‐sensitive MTH1 gene knockdown by the CRISPR‐Cas9 system significantly enhanced the therapeutic effect of SDT via sensitizing tumor cells to SDT and hindered the self‐healing function of tumor cells. In addition, Chen et al. connected the nanobubbles (NBs) with the sonosensitizer (indocyanine green [ICG]) and constituted an all‐in‐one nanoplatform carrying RAS plasmid (RSL3, ferroptosis promoter) (abbreviated into RSL3@O_2_‐ICG NBs) (Figure 4e). The experimental group RSL3@O_2_‐ICG NBs caused obvious decreases and damages of mitochondrial membrane potential of HepG2 and Huh7 cells compared to other groups (Figure 4f), suggesting the occurrence of ferroptosis. In contrast, ferroptosis inhibitor (Fer‐1) introduction resisted the GSH drop via blockading ferroptosis (Figure 4g,h). These experimental results show that RSL3@O_2_‐ICG NBs indeed realized the ferroptosis‐enhanced SDT.^[^ ^87^ ^]^ As the programmed cell death, apoptosis and ferroptosis are two dominant cell death pathways, and thus, the apoptosis or ferroptosis‐enhanced SDT is expected to enable the antigen exposures and presentations, which will further activate the systematic immune responses to favor the subsequent autologous immunotherapy and combined immunotherapy against tumor progression and metastasis.

#### 3.1.2 Nanobiotechnology‐Augmented SDT for Activating Immune Responses

**Figure 5:**
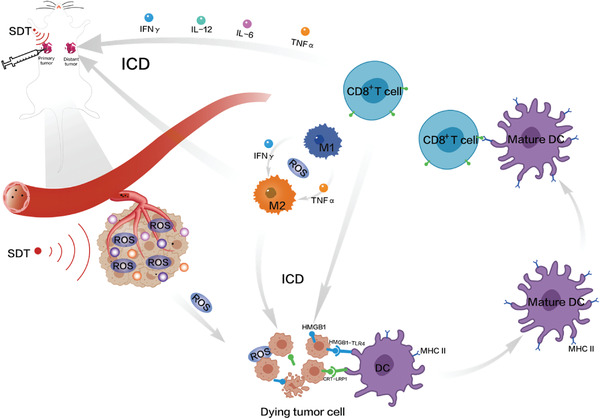
Principle schematic on the continuous inertial cavitation for promoting ROS production and enhancing ICD.

Besides directly oxidizing cell membrane and DNA, sonocatalytic ROS production in SDT could also potentiate ICD via releasing tumor‐associated antigens (TAAs), facilitating TAAs engulfment and presentation by antigen‐presenting cells (e.g., dendritic cells [DCs]), and activating T cells maturing and effector T cell infiltrations especially after combining with ultrasound‐targeted nanobubble destruction (UTND) technology.^[^ ^89^ ^]^ The nanobiotechnology or nanomedicine can boost ROS level through various pathways, wherein various nanoparticles that had been engineered into new nanosonosensitizers received increasing attentions.^[^ ^101^ ^]^ As a paradigm, Yin et al. designed continuous CO_2_ bubbling nanoreactors based on the reversible CO_2_ adsorption/desorption by amino groups, and the continuous CO_2_ release enabled the continuous inertial cavitation, which continuously and tremendously produced ROS.^[^ ^28^ ^]^ After directly killing tumor cells, the tumor cell fragments served as tumor antigens to induce the robust anti‐tumor immune responses and brought about massive ICD (**Figure** **5**). From the perspective of molecular level, the hallmarkers of ICD including adenosine triphosphate, high mobility group protein 1 (HMGB1), and calreticulin (CRT) were detected,^[^ ^102^ ^]^ and these indicators were highly expressed, promising the occurrence of ICD. The increases of DCs, T‐lymphocytes (CT8 ^+^ T cells, CTLs), and NK cells were upregulated and the infiltration of CD8 ^+^ T cells were found to rise in metastatic tumor tissues. In addition, cytokines such as tumor necrosis factor‐*α* (TNF‐*α*), interferon‐*γ* (IFN‐*γ*), and interleukin‐2 (IL‐2) were also activated. More significantly, the protumorgenic M2‐type macrophages were polarized into M2‐type ones, mitigating the ITM, which further strengthened the anti‐tumor immune actions.

The above strategies for elevating ROS from the perspective of rationally designing sonosensitizers were also believed to be utilized to potentiate the SDT‐activated immune responses. Typically, robust ICD of cancer cells can be induced by GSH depletion‐enhanced SDT especially after uniting with oxaliplatin (Oxa(IV)).^[^ ^90^ ^]^ In detail, Shen et al. synthesized a new multifunctional nano‐drug THPP Oxa (IV)‐PEG, which can release oxaliplatin in response to GSH. In other words, it can deplete GSH. By detecting the expression levels of typical ICD markers (CRT, HMGB1), it is proved that GSH depletion‐enhanced SDT as well as chemotherapy can effectively induce immunogenic cancer cell death. Concurrently, it was found that the numbers of mature DCs in inguinal lymph nodes and CD3 ^+^ CD8 ^+^ T cells and CD3 ^+^ CD4 ^+^ T cells in tumors were increased significantly, indicating that this treatment could activate the systematic anti‐tumor immune responses. Impressively, the effective immune memory effect was activated to prevent the recurrence of the tumor.

#### 3.1.3 Nanobiotechnology‐Augmented SDT in Combination With Immunotherapy

**Figure 6:**
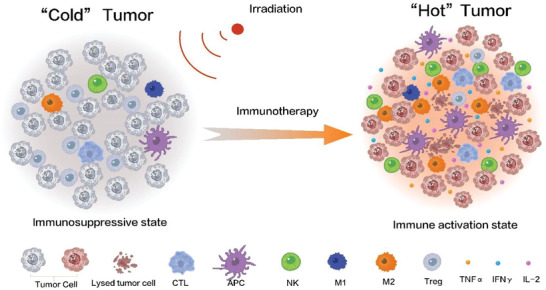
Illustration on SDT in combination with immunotherapy for synergistically activating and enhancing antitumor immunity.

Immunotherapy is different from traditional surgical resection, radiotherapy, and chemotherapy, and it relies on autoimmunity. Immunotherapy can kill tumor cells and tissues by activating or enhancing the human immune system, which thereby, plays a remarkable role in tumor prevention and can be regarded as the sole strategy for cancer eradication without relapse and metastasis.^[^ ^103^ ^]^ Effective immunotherapy can also promote the formation of memory immunity objective to tumor cells and exert the positive preventive actions on distant metastasis and recurrence of tumor.^[^ ^104^ ^]^ Inspiringly, the above advances of sonosensitizers driven by nanobiotechnologies can mitigate the ITM and activate and increase effector T cells infiltrations when high sonocatalytic activity is equipped to produce ROS in SDT process (**Figure** **6**). This pre‐treatment of nanobiotechnology‐augmented SDT that alters TME and promotes the transformation from a “cold” tumor in the immunosuppressive state to a “hot” tumor in the immune activation one,^[^ ^105^ ^]^ deserves to be exploited to contribute to the enhanced immunotherapy such as immune checkpoint blockade (ICB). To comprehensively understand it, the nanobiotechnology‐augmented SDT for activating anti‐tumor immune responses and combined therapy with immunotherapy is summarized in **Table** **1**.

**Table 1:** Names, compositions, and functions of different composite nanosonosensitizers and/or combined immunotherapy

Name	Composition	Model	Function	Refs.
TiO_2_‐Ce6‐CpG/aPD‐L1	TiO_2_, chlorin e6(Ce6), CpG ODN	Hepa1‐6	Enhanced SDT and immune response stimulation	^[^ ^62^ ^]^
Mn‐MOF	Mn‐MOF	4T1	Cyclooxygenase activity rise and GSH content drop	^[^ ^88^ ^]^
sPD‐1/Ce6‐NBs	Ce6, NBs	HCC	Induced UTND, enhanced SDT and immune response	^[^ ^89^ ^]^
RBC‐mTNPs@AQ4N	mTNPs, AQ4N, RBC	MCF‐7	Immune escape, hypoxia activation chemotherapy	^[^ ^63^ ^]^
THPP‐Oxa(IV)‐PEG	THPP, Oxa(IV)SA_2_, PEG_5k_‐COOH	CT26	Immunogenic nanodrug and radioisotope imaging	^[^ ^90^ ^]^
PIH‐NO	HSA‐NO, FDC, IR780	4T1	Mitochondrial damage, drug accumulation, and M2 macrophage polarization into M1 phenotypes	^[^ ^91^ ^]^
LiP‐PFH	DPPC, DSPC, DSPE‐PEG2000	4T1	Enhanced anti‐tumor immunity	^[^ ^92^ ^]^
OI_NPs	OXP, ICG, PFP	ID8	Improved optical and ultrasonic imaging	^[^ ^93^ ^]^
FA‐MnPs	MNP, FA	TNBC	M2 macrophages polarized to M1 phenotype, ICD response to activate NK, DC maturation	^[^ ^94^ ^]^
RSL3@O_2_‐ICG NBS	NBS, ICG, RSL3	HCC	Ferroptosis	^[^ ^87^ ^]^
SnNSs@PEG	2D SnNS, PEG	H1299	Excellent photothermal efficiency	^[^ ^64^ ^]^
MPN‐PALF	LOX, ATO, Ce6	4T1	Relieving acidic TME	^[^ ^95^ ^]^
MG@PNPs	PMnC, Gox	4T1	MR/PA dual‐mode imaging and tumor starvation treatment	^[^ ^96^ ^]^
*α*‐Fe_2_O_3_@Pt	*α*‐Fe_2_O_3_, Pt	4T1	Inhibiting electron‐hole pairs recombination for ROS production	^[^ ^97^ ^]^
P/M@CasMTH1	MOF, CRISPR‐Cas9, MTH1	A549	Controlled release and enhanced SDT	^[^ ^86^ ^]^
MitoCAT‐g	CAT‐g, CA, TPP	HCC	GSH exhaustion	^[^ ^84^ ^]^
Wettability of nanoparticles	MSN, PSS	Thrombus	Enhanced SDT	^[^ ^59^ ^]^
FA‐N‐GQds	FA, N‐GQD, pyridine N, pyrrole N	MDA‐MB‐231	High tumor labeling rate	^[^ ^61^ ^]^
ZIF‐8 NCS	ZIF‐8, MOF	4T1	Acidic TME modulation	^[^ ^80^ ^]^
IRO@FANP	PLGA, PEG, FA	ID8	Enhanced SDT	^[^ ^98^ ^]^
IR820@NCP	IR820, FTS	HCC	Tumor aggregation and fluorescence imaging	^[^ ^99^ ^]^
Pd/H‐TiO_2_NSS	Pd, NSS	C6	Enhanced CDT and SDT	^[^ ^100^ ^]^

**Figure 7:**
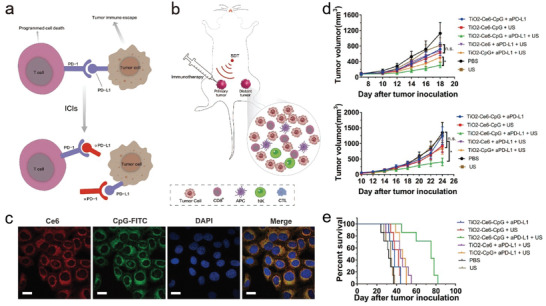
a) Principle illustration of immune checkpoint inhibitors for promoting the recognition of tumor cells by T cells and restoring the number of T cells in tumors. b) Operation and mechanism schematic on SDT combined with immunotherapy for repressing the primary tumors and distant tumors on the bilaterally‐implanted tumors, where an increase in the proportion of immune cells in the distant tumor was observed. c) CLSM images of Hepa1‐6 cells after incubation with nanosonosensitizers. d) Time‐dependent growth profiles of primary (left) and distant (right) tumors in different groups. e) Survival curve of treated mice in different groups. Reproduced with permission.^[^ ^62^ ^]^ Copyright 2021, Dove Press Lt.

ICB immunotherapy has become the first‐line treatment choice for multiple solid tumors (**Figure** **7**a), but the clinical benefit rate is limited, ranging from 10% to 40% and only a small portion of patients respond to ICB, which severely limits their clinical application.^[^ ^106^, ^107^ ^]^ In this regard, combined therapy with various therapeutic methods is usual and has gained a large number of explorations.^[^ ^108^, ^109^, ^110^ ^]^ Especially, the combination of SDT with ICB is burgeoning, and Lin et al. studied SDT combined with immune checkpoint inhibitor aPD‐L1 (TiO_2_‐Ce6‐CpG/SDT/aPD‐L1) based on nanosonosensitizer (TiO_2_‐Ce6‐CpG). This combined therapy enabled the activation and maturation of DCs, the infiltration of CD8 ^+^ T cells in tumor tissues, and the immune escape target (PD‐L1) blockade (Figure 7b).^[^ ^62^ ^]^ In vitro experiments revealed that TiO_2_‐Ce6‐CpG showed an efficient internalization into the cells (Figure 7c). After various experimental analysis, it was found that CD11c and CD86 as the representative markers of DCs maturation were up‐regulated, indicating that nanosonosensitizer TiO_2_‐Ce6‐CpG could significantly promote DCs maturation in vitro. In the in vivo experiment of female C57BL/6 mice inoculated with Hepa1‐6, it was found that the treatment with such composite sonosensitizers (TiO_2_‐Ce6‐CpG) combination with PD‐L1 inhibitor could not only effectively inhibit the primary tumor but also significantly delay the growth of distant tumors (Figure 7d). Moreover, the survival time of mice in the experimental group was significantly prolonged (Figure 7e). In such combined therapy of SDT with ICB, immunologic adjuvants play an important role associated with immunity promotor.^[^ ^108^ ^]^ Yue et al. synthesized a liposomes‐supported SDT nanoplatform co‐loading sonosensitizers and immunologic adjuvants, where the strong immune responses and immune memory effects were activated to repress primary and distant tumors and rechallenged ones.^[^ ^111^ ^]^ Further, genetics regulation was harnessed to assist the ultrasound‐guided immunotherapy especially after integrating with nanobiotechnology. Typically, Li et al. designed ultrasound‐guided immunotherapy nanocomplexes,^[^ ^112^ ^]^ wherein the nanocomplexes were obtained based on ACPs‐targeting microbubbles that were coated with 2′3′‐cyclic guanosine monophosphate‐adenosine monophosphate (cGAMP)‐adsorbed biopolymers. Through binding APCs, the loaded cGAMP was readily delivered into cytosol via microbubble cavitation‐enhanced membrane permeability to activate interferon‐related cGAS‐STING pathway and proinflammatory pathways and further stimulate antigen‐specific T cells, which successfully connected innate and adaptive anti‐tumor immunity to repress local and metastasized tumors especially after combining ICB immunotherapy.

In brief, SDT based on nanomaterials can effectively activate the systematic anti‐tumor immune responses, which could not only delay the primary tumors and produce remarkable therapeutic effects against metastatic tumors but also actively prevent tumor recurrence.^[^ ^113^ ^]^ It is believed that SDT combination with immunotherapy will make large breakthroughs as nanobiotechnology moves forward.

#### 3.1.4 Nanobiotechnology‐Enhanced HIFU Ablation for Activating Immune Responses

High‐intensity focused ultrasound (HIFU) as one of the prevalent tumor thermal ablation means is recognized all over the world to treat various cancers, such as liver cancer, kidney tumor, pancreatic cancer, and prostate cancer and also to relieve the pain of tumors.^[^ ^114^, ^115^ ^]^ HIFU plays the thermal and mechanical roles in the tumor ablation. The thermal effect can make the local tissue temperature rise to 60 °C rapidly and then induce tissue coagulative necrosis, which is the main cause in HIFU ablation treatment.^[^ ^116^ ^]^ The treatment methods of HIFU include ultrasound‐guided high‐intensity focused ultrasound (USgHIFU) and magnetic resonance‐guided high‐intensity focused ultrasound (MRgHIFU). USgHIFU uses diagnostic ultrasound to locate the target area and observe the treatment response in real‐time.^[^ ^117^ ^]^ With regards to MRgHIFU, Deng et al. constructed pluronic polymer and Gd‐DTPA‐modified MSN (Pluronic‐Gd‐MSN) nanoparticles, and the obtained nanoparticles could respond to HIFU, change local T1 (spin‐lattice relaxation time), and enhance image contrast. Under MRI guidance, the focused ultrasound transducer could make their focus on tissue sites as small as 1.5 mm^3^ for treatment.^[^ ^118^ ^]^ MRgHIFU also provided a noninvasive real‐time temperature measurement function, which accurately controlled temperature and heat dose for ablating deep bone metastasis.^[^ ^119^ ^]^

Thermal ablation can effectively activate a human anti‐tumor immune response.^[^ ^120^ ^]^ The activated immunity mechanism is summarized as follows: 1) Tumor fragments and related antigens after HIFU treatment served as tumor vaccines to enhance the anti‐tumor immunity. 2) HIFU induced Th1 response and enhanced the activity of DCs and CTLs. 3) HIFU balanced the immunosuppressive TME.^[^ ^121^ ^]^ It has been reported that the average value of NK cells, CD3 ^+^ T cells, CD4 ^+^ T cells, and CD4 ^+^/CD8 ^+^ T cells in the peripheral blood of patients with advanced pancreatic cancer after HIFU treatment were all increased.^[^ ^116^, ^122^ ^]^ After USgHIFU treatment was administered in 100 patients with pancreatic cancer (PaC) and in 30 patients with uterine myoma (UF), the early subclinical systemic inflammatory responses were triggered as serum LDH, leukocyte, C‐reactive protein (CRP), and IL‐6 were increased. The increased LDH indicated the protein release in cells, and the increase of leukocyte and CRP denoted the beginning of inflammatory responses in vivo. The increase of IL‐6 may be caused by the enhancement of macrophage and T‐lymphocyte activity in the acute stage. These results indicated that the early inflammatory environment of the whole body caused by HIFU treatment was the precondition for anti‐tumor immunity.^[^ ^123^ ^]^ In a research hosted by Zhu et al., 25 of 48 breast cancer patients underwent a modified radical mastectomy and another 23 patients underwent HIFU radical resection. After HE and immunohistochemical analysis of axillary lymph nodes, HIFU was observed to stimulate the immune responses of the whole body and significantly increase the number of effector T cells, activated CTLs, and NK cells.^[^ ^124^ ^]^

**Figure 8:**
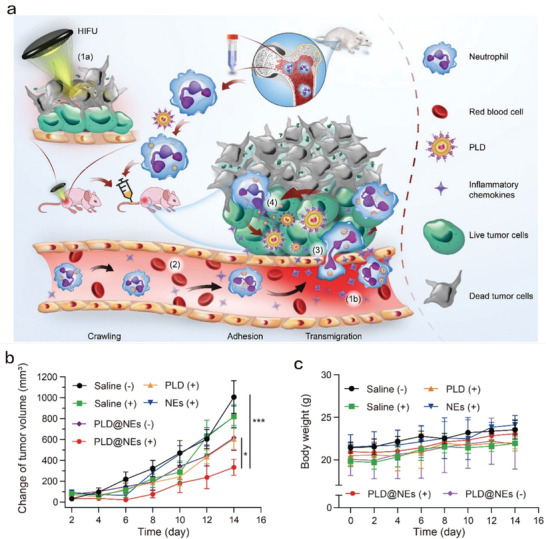
a) Schematic diagram of PLD‐loaded NEs for inhibiting tumor recurrence after HIFU treatment. b) The time‐related variation profiles of tumor volume relative to initial volume in each experimental group. c) Body weight change of mice in each experimental group. Reproduced under the terms of the Creative Commons CC‐BY license.^[^ ^130^ ^]^ Copyright 2021, The Authors. Published by Springer Nature.

Although many benign tumors can be treated with HIFU,^[^ ^123^ ^]^ the coagulation necrosis caused by thermal effect is unsatisfactory in inducing immune responses.^[^ ^125^, ^126^, ^127^ ^]^ To address it, combined therapy with other treatment methods is highlighted to magnify ablation outcomes for potentiating the immune responses after HIFU treatment. Notably, chemotherapy was employed to combine with HIFU to enhance ablation consequence and activate the systematic immune responses to further augment immunotherapy. In the treatment of triple‐negative breast cancer (TNBC), HIFU was combined with gemcitabine (GEM) to treat TNBC (4T1 tumor) in mice. Results showed that the combined therapy significantly inhibited the TNBC tumor, prolonged the overall survival time of mice, and proved that the inhibition of tumor was related to the increase of T cells.^[^ ^128^ ^]^ Another study proved that MRgHIFU could repolarize tumor‐related macrophages (TAM) into M1 subtypes and enhance the infiltration of T cells in the TNBC model. As they were used in mouse neuroblastoma, immune escape blockade using aPD‐L1 and mAb was implemented after HIFU ablation, and they cooperatively caused the potent anti‐tumor immune responses of the whole body and reduced the recurrence and metastasis of advanced tumors.^[^ ^120^, ^125^ ^]^ Similar results were obtained in the mouse pancreatic cancer model.^[^ ^126^ ^]^ PEI‐PLGA‐NaHCO_3_‐NPs developed by the Wang et al. showed an effective tumor‐targeting effect in the 4T1 breast cancer model, which was much preferable for enhancing HIFU treatment.^[^ ^129^ ^]^ Intriguingly, Shen et al. used neutrophils as carriers to entrap and encapsulate PEGylated liposome adriamycin (PLD) nanoparticles to establish the composite therapeutic drug (PLD@NES) (**Figure** **8**a).^[^ ^130^ ^]^ With Hepa1‐6 cells and corresponding tumor‐bearing mouse models, in vivo experiments showed that PLD@NES could effectively target and remove the residual tumor tissues after HIFU ablation with higher efficiency and lower side effects (Figure 8b,c), consequently receiving the most robust anti‐tumor consequences. Comprehensive immune characterizations revealed that the mechanical stress deriving from HIFU could promote ICD of human breast cancer cells through tumor necrosis‐associated signal pathways, which significantly increased the damages‐related molecular patterns (e.g., CRT, HSP70, and HMGB‐1) and inflammatory cytokines (IFN‐*γ*, IL‐1*α*, IL‐1*β*, and IL‐18), and drove the secretion of chemokine (IL‐8) relating to M1 macrophage activation.^[^ ^131^ ^]^

Besides combined therapy, enhanced energy utilization efficiency using various methods is also expected to make massive antigens exposure and activate immune responses for repressing tumor. In light of the fact that cavitation will elevate energy utilization efficiency and induce ICD,^[^ ^79^ ^]^ exogenously artificial cavitation nuclei were also introduced to reinforce HIFU ablation‐based anti‐tumor outcomes.^[^ ^132^, ^133^, ^134^, ^135^ ^]^ DL‐menthol (DLM)‐based and various microbubbles‐based cavitation nuclei have been constructed to realize it. Especially, DLM‐based cavitation nuclei featured multiple enhancements under one injection,^[^ ^134^ ^]^ which completely matched the pulsed and multi‐point operations of HIFU ablation, determining that DLM‐based cavitation nuclei outperformed micro‐ or nano‐sized bubbles cavitation nuclei in boosting energy utilization efficiency and ablation outcomes.

Based on acoustic cavitation, histotripsy ablation was developed to destroy tissue (M‐HIFU), which could produce an adaptive immune response to a tumor in spite of no thermal effects. A lot of of evidence shows that histotripsy activation could stimulate the specific immune responses objective to tumor and further amplify the immunotherapy effect of the checkpoint inhibitor.^[^ ^136^, ^137^ ^]^ Under the help of nanotechnology and nanomaterials, Li et al. bridged a contrast agent and a chemotherapy drug to engineer smart nanodroplets and such nanodroplet exhibited the M‐HIFU/GSH dual‐responsive drug release property. Experimental results showed that the combined multiple effects activated DCs maturation, induced robust ICD, and enhanced the infiltrations of effector T cells to effectively inhibit tumor growth, providing an effective method for tumor treatment of nanoparticles combined with HIFU.^[^ ^114^ ^]^ Liquid fluorocarbon‐encapsulated nanobubbles or nanoemulsion as cavitation nuclei could further enrich the functions. Typically, perfluorooctane bromothane (PFOB)‐based nanoemulsion could be engineered as vehicles to load MnO_2_ nanoparticles (MBP)^[^ ^138^ ^]^ and simultaneously retained the dual‐mode MRI/CT imaging functions as PFOB and MnO_2_ could serve as CT and MRI contrast agents, respectively. It is worth noting that MnO_2_‐mediated Fenton‐like reaction could consume GSH, which along with PFOB cavitation‐enhanced HIFU and its activated immune responses, contributed to the significantly‐increased ICD and eventually enhanced the inhibitory effects against tumor growth and lung metastasis.

### 3.2 MWA Treatment

MWA is still considered a local treatment for tumors. It emits a microwave oscillating electric field through a needle, which greatly induces temperature rise and coagulation necrosis of tumor tissues of interest, thus, reducing the tumor burden. Nowadays, it has become a reliable local treatment for various solid tumors, for example, liver cell carcinoma, renal cell carcinoma, lung cancer, and bone tumor.^[^ ^139^, ^140^, ^141^ ^]^ Xu et al. reported a case with multiple lung metastases of endometrial cancer. After chemotherapy and endocrine therapy were ineffective, the focus underwent MWA. Results showed that the metastases gradually disappeared, and no new metastases were found during the following 3‐year follow‐up.^[^ ^142^ ^]^ According to the medical record report by Wei et al., a 47‐year‐old and a 55‐year‐old female patient with non‐small cell lung cancer received camrelizumab administering (200 mg every 2 weeks) one week after MWA. After four treatment cycles, the tumor on the 47‐year‐old patient treated with MWA reached complete ablation, and the metastatic tumor reached complete response, Similarly, the primary lesion of the 55‐year‐old patient was completely ablated, and other lesions showed partial response (PR).^[^ ^143^ ^]^ Compared with other ablation methods such as RFA, MWA can generate higher temperature (more than 100 °C), larger ablation volume, and shorter ablation time.^[^ ^144^ ^]^ In addition, MWA is also suitable for larger tumor treatment because it is almost not affected by the change of tissue impedance and can effectively pass through fibrous tissues. Compared with other thermal ablation techniques, MWA can rapidly heat tissue and is insusceptible to the heat sink effect.^[^ ^145^, ^146^ ^]^ However, akin to RFA, MWA is usually used as a single treatment, accompanied with the postoperative tumor recurrence rate remaining high, and the prognosis is unsatisfactory. Therefore, it is necessary to develop new solutions to overcome these concerns. In an attempt to address them, MWA sensitizers and combined therapy with other treatment methods for expanding MWA outcomes as much as possible were prevalent.

#### 3.2.1 MWA‐Activated Immune Responses

It is also reported that MWA treatment could inhibit the metastasis of primary breast cancer via activating the macrophage/IL‐15/NK cell axis. In this study, two types of stage‐IV breast cancer mice models were used to determine whether MWA could inhibit lung metastasis of breast cancer and improve the survival rate of mice. Robust evidence revealed that macrophages were activated and IL‐15 was produced after MWA treatment, and NK cells were instigated to inhibit tumor metastasis.^[^ ^144^ ^]^ Zhou et al. found that MWA could activate the ICOS pathway to induce acquired immunity in the treatment of breast cancer.^[^ ^147^ ^]^ It was also found that the proinflammatory cytokines including IL‐1*β* and IL‐6 were increased in the circulatory system after MWA treatment, suggesting the occurrence of inflammatory responses.^[^ ^148^ ^]^ Another team used the single‐cell sequencing technology to analyze and compare the peripheral blood mononuclear cells (PBMCs) of six patients with early breast cancers before and after MWA treatment. Macroscopically, MWA treatment was found to induce systemic immune responses and confirmed that B cells played an important role in MWA‐activated immune responses.^[^ ^149^ ^]^

The relationship between the specific immune response and T‐cell response and tumor treatment outcome was analyzed in the study conducted by Katharina et al. who traced and assayed their levels in the peripheral blood of patients with liver cancer after MWA.^[^ ^150^ ^]^ Several results were obtained, for example, 1) The TAAs for specifically activating T cell responses were enhanced after MWA. 2) After MWA, the activated T cells and B cell subsets were increased in patients with long‐term remission. 3) The antigen‐specific T‐cell responses after MWA gave rise to a long disease‐free survival period. The phenomenon disclosed that MWA had a significant anti‐tumor immune correlation effect in patients with liver cancer and provided reliable evidence for the combination of local MWA and immunotherapy. In addition, combining MWA with TIGIT blockers may be a potential clinical treatment strategy. In Chen et al.’s study, the expressions of anti‐human t‐globulin (ATG) and anti‐tumor module domain (TIGIT) based on tyrosine were prominently upregulated after MWA. The combination of TIGIT blocker and MWA significantly promoted the increase of CD8^+^ TILs in tumor. These results showed that TIGIT blocker introduction in MWA could up‐regulate the expressions of CXCL9 and CXCL10 in TAMs, boost CXCR3 expression in T cells, and promote CD8^+^ TILs activation and infiltration, which may inhibit tumor growth and enhance anti‐tumor immunity.^[^ ^151^ ^]^ Furthermore, to explore the mechanism, some scholars used image‐guided ablation (IGA) to analyze the plasma protein profile and shed light on the principle including molecular signaling pathway after small renal tumor treatment. One hundred sixty‐four circulating proteins in the blood of patients with small renal tumors treated by ablation were analyzed to determine the immune effects on body system and to understand ablation effects on the tumor and immune system. This study provided fundamental information for the design of MWA in combination with immunotherapy.^[^ ^152^ ^]^

#### 3.2.2 Nanobiotechnology‐Enabled MWA Sensitizers for Activating or Potentiating Immunity

MWA nanosensitizers based on nanobiotechnology are an important direction of activating immune responses via magnifying MWA outcomes with lowering MWA power or time. Usually, exogeneous cavitation nuclei are common for enhancing MWA via inertial cavitation‐mediated energy transformation, akin to those in HIFU. As the nanotechnology proceeded, great efforts have been focused on metal‐based nanoparticles, where the enriched ions’ frication with each other are activated and ion oscillation in the presence of MWA field is induced, both of which are responsible for the heat elevation.^[^ ^153^, ^154^, ^155^ ^]^

**Figure 9:**
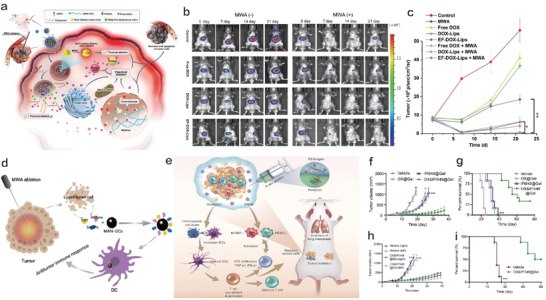
a) The illustration on the physical and chemical abation actions of such non‐ionic MWA nanosensitizers for improving the conversion efficiency from electromagnetic energy to heat and increasing the sensitivity of MWA. b) In vivo fluorescence images and c) quantitative signal intensity circled at the site of tumor as a function of time. Reproduced with permission.^[^ ^156^ ^]^ Copyright 2022, American Chemical Society. d) The schematic image of MWA in combination with nanoparticles (MAN‐DCs) for activating the anti‐tumor immune response in vivo. e) Application diagrams of OX&IPI549@Gel bioscaffold‐enabled chemoimmunotherapy in inhibiting tumor growth and metastasis after incomplete microwave ablation. f) Tumor growth curves and g) mouse survival rates in different experimental groups. h) Volume variation curves of primary tumor and distant tumor and i) the survival rate of mice in each experimental group. Reproduced with permission.^[140^ ^]^ Copyright 2021, American Chemical Society.

To overcome the shortcomings of current exogeneous cavitation nuclei and metal‐based nanoparticles for enhancing MWA, that is, uncontrollable and nonpersistent enhancement actions and inevitable ion disruption‐enabled dyshomeostasis of normal tissues, respectively, biocompatible non‐ionic MWA nanosensitizers were constructed.^[^ ^156^ ^]^ In this non‐ionic MWA nanosensitizer, polar but non‐ionic ethyl formate (EF) molecules as the liquid inner core were successfully loaded in liposomes along with doxorubicin (DOX). EF with high volatility could realize three enhancement actions (**Figure** **9**a), that is, EF bubbles‐mediated cavitation, polar oscillation‐mediated electricity‐heat energy conversion, and EF metabolism‐enabled chemical ablation after degradation into ethanol. The three actions were sufficiently validated and leveraged to enhance MWA outcome, which further cooperated with released DOX to perform the best in repressing tumor progression and recurrence of multiple orthotopic nodules (Figure 9b,c). Mechanistic analysis revealed that the excellent anti‐tumor outcome was attributed to the systematic immune activations and ITM mitigation induced by the three actions, including the increased infiltrations of CD8^+^ CTLs, NK cells, and memory CD3^+^ T cells; boosted anti‐tumor cytokine secretions (TNF‐*α* and IFN‐*γ*); and decreased myeloid‐derived suppressor cells (MDSCs) in tumor. Zhou et al. conducted an interesting work, and they developed mannose‐derived carbon dots (MAN‐CDs) nanoparticles on the basis of the fact that MWA can dissolve tumor cells and release cell fragments containing tumor‐related antigens. Although the introduced nanoparticles failed to significantly enhance MWA, they could effectively capture many “danger signals” (DSs) after MWA and present these DSs to DCs so as to induce the body to produce strong anti‐tumor immune responses. The treatment protocol could not only inhibit the growth of primary tumors and distant tumors but also brought about the long‐term immune memory effects to eliminate the re‐attacked tumor cells (Figure 9d).^[^ ^157^ ^]^ The two cases referring to activated or enhanced immune responses using their corresponding MWA immune promoters lay a solid foundation to subsequent combined therapy of MWA with immunotherapy.

#### 3.2.3 Nanobiotechnology‐Enabled MWA‐Combined Immunotherapy

There is increasing evidence that MWA can not only physically eliminate tumors but also play a therapeutic role in the location of metastatic lesions especially after combining with immunotherapy.^[^ ^158^ ^]^ In a case report study, a 69 year old patient with metastatic squamous lung cancer was reported. Despite generating the immunotherapy resistance, the clinical treatment results showed that MWA produced the durable ablation consequences. This phenomenon found that local ablation not only eliminated the primary lesion but also inhibited the metastatic lesion by enhancing the systematic immune responses.^[^ ^159^ ^]^

Shen et al. found that the immunosuppression enhancement mediated by bone marrow cells after incomplete microwave ablation (iMWA) was the main cause of high invasiveness that residual tumors were confronted with. In order to solve this issue, they used phosphatidylinositol 3‐kinase*γ* (PI3K*γ*) inhibitor (IPI549) to target bone marrow cells and concurrently, oxaliplatin (OX) was combined in hydrogels after MWA (Figure 9e).^[^ ^140^ ^]^ The injectable hydrogels usually served as the reservoir of drugs and cells, which can magnify local treatment outcomes of various lesions.^[^ ^160^, ^161^, ^162^, ^163^ ^]^ Such OX&IPI549@Gel treatment significantly inhibited tumor growth after iMWA on colorectal cancer (CT26)‐bearing mice model (Figure 9f,g). Intriguingly, MWA combined with the chemotherapy and immune activation promoter could provide long‐term immune memory function, protect mice from the re‐attack of tumor cells after eliminating the initial tumor, and also wake the strong anti‐tumor immune responses to inhibit metastasis and proliferation (Figure 9h,i).

MWA combination with anti‐PD‐1 treatment‐represented ICB can inhibit the growth of distant tumors, establish a systematic anti‐tumor immune environment, and reduce recurrence. Studies have shown that the combined therapy of MWA with anti‐PD1 (aPD‐1)/CTLA‐4 could prolong the survival time of mice and prevent tumor recurrence in the hepatocellular carcinoma mouse model.^[^ ^139^ ^]^ The main mechanism unraveled was that MWA activated T cell immune responses against hepatoma cells and enhanced the intratumoral infiltration of cytotoxic T lymphocytes (CTLs) induced by MWA. The combined treatment also enhanced the immune response of Th1 cells, reduced Th2 cytokines, led to Th1 cell polarization, and displayed the specific antitumor immunity. In the study conducted by Huang et al., a hepatocellular carcinoma model was established in mice and then treated with MWA combined with aPD‐1.^[^ ^141^ ^]^ They showed that the survival period of the combined group was longer than that of MWA or aPD‐1 alone, and the distant tumor growth was considerably delayed. In addition, the MWA&aPD‐1 combined therapy improved the level of Th1 cytokines in peripheral blood and showed a stronger ability to induce a specific anti‐tumor immune response than the treatment of aPD‐1 or MWA alone. These results unveiled that MWA combined with immunotherapy could induce and enhance the anti‐tumor immune responses, thus, reducing the recurrence rate and improving the survival rate of tumor patients.

### 3.3 RFA Treatment

Local ablation including RFA and MWA is the first choice for the treatment of early HCC,^[^ ^158^ ^]^ and RFA is another minimally‐invasive and local treatment means.^[^ ^59^ ^]^ RFA is driven by a high‐frequency electric pulse to produce radio frequency waves and then heat tumor.^[^ ^164^ ^]^ The electricity‐heat conversion principle depends on the friction motion of ions under the action of high‐frequency oscillation current, and the heat effect can make the temperature at the central region reach above 60 °C. The high temperature leads to the solidification necrosis of tumor tissues around the electrode,^[^ ^165^ ^]^ enabling RFA to become the main ablation method for early HCC with a diameter of less than 5 cm.^[^ ^166^, ^167^ ^]^ Similar to other treatment methods, the eradication of tumor needs the participation of activated autoimmunity or combined therapy‐potentiated immunity.

#### 3.3.1 RFA‐Activated Immune Responses

RFA can induce a large number of heat solidification necrosis by radiofrequency wave and produce corresponding antigens, which induce tumor‐specific T cell response. At the same time, it can further induce the adaptive immune response to the tumor and treat the primary tumor and distant metastasis tumor.^[^ ^168^ ^]^ In detail, RFA can produce a large number of cell fragments during the apoptosis and necrosis of cancer cells. These damaged tumor cells may provide impetus to induce tumor immunity in vivo, which can produce immune responses by promoting the maturation of APCs. The current evidence shows that RFA not only has a positive effect on tumor immunity at a local level but also triggers systemic immune responses.^[^ ^166^ ^]^ In brief, RFA has a better therapeutic effect, good safety, and few complications, similar to the vaccines.

In the oligometastasized breast cancer, RFA therapy benefits oligometastases recession,^[^ ^169^ ^]^ and in colorectal cancer lung metastasis, the invasive rate of RFA treatment was much lower than that of surgical resection.^[^ ^170^ ^]^ Despite this, according to clinical experiences, with RFA, it is very difficult to treat large tumors (>5 cm) because the adequately‐high temperature field is difficult to cover the whole tumor and results in incomplete RFA. In addition, cancer patients with multiple site metastasis (*n* > 3) are also found difficult to appropriate for RFA.^[^ ^164^ ^]^ Moreover, the low energy conversion efficiency in part contributes to the incomplete RFA.^[^ ^79^, ^171^ ^]^ Therefore, RFA as a single treatment alone fails to induce sufficient immune responses to eliminate residual cancer cells and avoid tumor recurrence. More significantly, the incomplete RFA has been validated to inevitably pose local recurrence and ITM potentiation and heighten the tumor resistance to immunotherapy, consequently resulting in the failure of combined immunotherapy and accelerating tumor metastasis.^[^ ^172^ ^]^ Inspiringly, the marriage of RFA and immunosuppressants can significantly evoke the T cell response toward tumor antigens and inhibit the growth of distant tumors, which is expected to lower the recurrence and metastasis rates of incomplete RFA.^[^ ^168^ ^]^

#### 3.3.2 Nanobiotechnology‐Enabled RFA Enhancers for Activating or Potentiating Immunity

Akin to MWA, EFA enhancer that could elevate the energy utilization efficiency and improve RFA outcome for activating and magnifying immunity also deserves expectation to resist incomplete RFA and repress tumor progression, relapse, and recurrence via activating immune responses. In the design of RFA enhancers, magnetic nanoparticles (MNPs) are the most prevalent one because they could mediate the magnetothermal conversion under the alternating electromagnetic field during RFA to improve the energy utilization efficiency. Depending on the high energy conversion property, cavitation effect was introduced and qualified to serve as the foundation of RFA enhancer design. To realize the continuous inertial cavitation, Zhang et al. developed a radiofrequency solidoid vaporation (RSV) strategy based on DLM, and they utilized polylactic acid glycolic acid (PLGA) carriers to encapsulate DLM.^[^ ^173^ ^]^ The obtained DLM@PLGA nanocapsules could respond to RFA‐unlocked heat and trigger the continuous solid–liquid–gas tri‐phase transition of DLM, eventually harvesting the largest RFA volume with descended power and time. Furthermore, chemotherapeutic drugs were co‐loaded in PLGA carriers along with DLM and then active targeting ligands were chelated,^[^ ^174^ ^]^ which realized the targeting enhancement of RFA and combined therapy with chemotherapy for resisting tumor progression.

**Figure 10:**
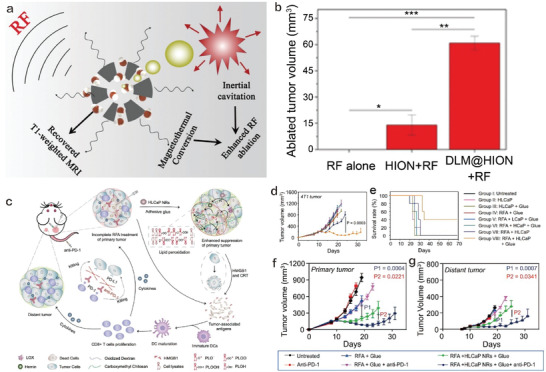
a) The principle schematic on DLM@m‐Fe_3_O_4_ for greatly improving the efficiency of RFA via DLM‐enhanced inertial cavitation and m‐Fe_3_O_4_‐unlocked magnetothermal conversion and eventually enabling the RFA monitoring and outcome assessment. b) Ablation volume of 4T1 tumor after treatment in each experimental group. Reproduced with permission.^[175^ ^]^ Copyright 2019, American Chemical Society. c) Mechanism illustration of enhancing RFA efficacy and antitumor immune response by tumor debris fused tumor‐killing HLCaP NRs. d) Tumor growth curve and e) mouse survival curve of different experimental groups. Tumor growth curve of f) primary tumor and g) distant tumor. Reproduced under the terms of the Creative Commons CC‐BY license.^[164^ ^]^ Copyright 2021, The Authors. Published by Springer Nature.

To further elevate the energy utilization efficiency, the magnetic Fe_3_O_4_ nanoparticles‐mediated magnetothermal conversion and DLM‐enabled continuous cavitation were integrated (**Figure** **10**a).^[^ ^175^ ^]^ Mesoporous Fe_3_O_4_ (m‐Fe_3_O_4_) that played the roles of magnetothermal conversion agents and containers to load DLM were used to engineer the composite DLM@m‐Fe_3_O_4_. Thanks to the combined actions, DLM@m‐Fe_3_O_4_ outperformed m‐Fe_3_O_4_ alone and DLM alone in augmenting RFA volume (Figure 10b). More significantly, the DLM tri‐phase transition made m‐Fe_3_O_4_ exposed away from DLM and altered the DLM‐arisen hydrophobic microenvironment of m‐Fe_3_O_4_ into hydrophilic one, which successfully varied the longitudinal relaxations and MRI signal and eventually enabled the RFA monitoring and outcome assessment.

#### 3.3.3 Nanomedicine‐Enhanced RFA‐Combined Immunotherapy

Immunotherapy in combination with local treatment such as RFA is believed to be a direction to control the progress of early liver cancer.^[^ ^176^ ^]^ Compared with the combination therapy, the single ICB shows limited clinical benefits.^[^ ^177^ ^]^ The combination of immunotherapy and other therapies elongated the overall survival (OS) and progression‐free survival (PFS). A clinical study showed that 59 patients with stage I or II liver cancer received adjuvant autologous cytokine‐induced killer (CIK) cell immunotherapy after radical resection or RFA, and the recurrence‐free survival (RFS) was significantly prolonged.^[^ ^178^ ^]^ In recent years, significant breakthroughs have been made in the combined therapy of RFA and immunotherapy. It is extensively accepted that RFA can destroy tumor cells and enhance anti‐tumor immunity by releasing tumor antigens from the killed tumor cells, and immunosuppressants (ICIs) can enhance the antitumor immune response induced by local therapy.^[^ ^179^ ^]^ Thereby, the two actions synergize to activate and potentiate anti‐tumor immunity in different dimensions such as time and space.^[^ ^180^ ^]^ There is lots of evidence that the combination of locoregional treatment (LRT) and ICB can produce synergistic effects on tumor immune response.^[^ ^165^, ^181^ ^]^ Tremelimumab is a human anti‐CTLA‐4 monoclonal antibody and it can improve its efficacy by combining RFA. In the treatment of local advanced pancreatic cancer, immunotherapy combined with RFA can increase the blocking activity of the checkpoint and the presentation of antigens to the immune system.^[^ ^182^ ^]^

Huang et al. treated hepatoma mice with aPD‐1 immunosuppressant and RFA in a preclinical model study. The results showed that the tumor decreased significantly in the combined treatment group, and the tumor‐infiltrating lymphocytes increased significantly, validating that this combination therapy exerted a significant antitumor effect.^[^ ^183^ ^]^ Animal experiments confirmed that the immune response mediated by T cells increased significantly after RFA.^[^ ^184^ ^]^ RFA could destroy primary liver cancer, stimulate the release of tumor antigens, and increase immune responses, which indicated that RFA in combination with immunotherapy was a promising new treatment method. Some studies used anti‐PD‐1 and anti‐CTLA4 antibodies to treat residual tumor after RFA‐mediated tumor size reduction. This dual‐targets blockade strategy restored the antitumor immunity of HCC by increasing antitumor T cell responses so as to prevent tumor recurrence and progression.^[^ ^166^ ^]^

To some extent, nanomedicine can overcome the limitations of current tumor treatment and provide more distinctive insights into tumor treatment.^[^ ^185^ ^]^ On the one hand, nanoparticles can maximize the delivery of genes, imaging units, or chemotherapy drugs; and on the other hand, the modified nanoparticles can spontaneously aggregate into tumor tissue and release killer substances so as to achieve the purpose of targeted tumor cell killing.^[^ ^31^, ^32^, ^45^, ^134^ ^]^ In recent years, researchers have proposed a new anti‐cancer treatment method which applies external physical stimulations and nanoparticles at the same time. The synergistic effect succeeds in inducing the apoptosis of cancer cells and reducing the damages to surrounding normal tissues.^[^ ^186^ ^]^ Yang et al. encapsulated the tumor‐killing HLCaP nanoreactor (NRs) prepared by lipoxygenase and heme in PLGA copolymer (Figure 10c).^[^ ^164^ ^]^ After RFA, the obtained nanomaterials inhibited the residual tumor by triggering ferroptosis and improved the efficacy of RFA. As an immunogenic nano‐drug, it could induce robust anti‐tumor immune responses and produce strong anti‐tumor effect (Figure 10d,e) so as to inhibit the further development of primary and distant tumors (Figure 10f,g). Taking all above together, RFA enhancers that can promote the ablation outcomes are designed to favor more antigen exposures and systematic immune response activation and to strengthen immunotherapy, which will arouse more interest in the near future.

## 4 Conclusions and Challenges

Compared with traditional tumor treatment protocols, SDT, HIFU, RFA, MNP, and MWA combined immunotherapy that served as a new treatment scheme which can release tumor‐specific antigens as they lyse tumor tissues. The released antigens further activate and enhance the body's anti‐tumor immune responses, change the tumor microenvironment, promote the transformation of TME from “cold” to “hot”, and eventually inhibit tumor recurrence and metastasis. In this process, nanomedicine shows great potential for adjuvant therapy as it could potentiate the physical treatment‐activated immunity. In addition, other functions such as nanomaterial‐assisted imaging could be added to achieve highly precise treatment. Different surface modifications of nanomaterials can not only significantly enhance the outcomes of corresponding treatment modes (e.g., SDT) but also realize the immune escape blockade and targeted delivery of drugs in vivo, and even achieve the controllable release of drugs in an on‐demand manner. In a word, the development of nanomedicine built a bridge for the combined treatment of different medical means. It is believed that the progressive development of different treatment methods such as ultrasound therapy, immunotherapy, and ablation therapy in the near future, will also provide concerned points to propel nanomedicine development. Despite acquiring massive achievements, there have many concerns that are yet to be investigated. First, the synergistic mechanism of nanomaterials, clinical physical treatment, and immunotherapy remains to be clear. The early detection using immune checkpoint inhibitor biomarkers to predict clinical benefits needs to be explored. Personalized treatment, multidisciplinary treatment, and optimal treatment strategy determination according to the immune specificity of each patient is still challenging but desirable. Besides the rational design of nanomedicine in terms of enhancing physical treatment outcomes and boosting immune responses, biocompatibility is another concern that should be taken into serious account.

## Conflict of Interest

The authors declare no conflict of interest.

## Author Contributions

Q.L. and W.Z. contributed equally to this work. *Conceived this theme, organized the structure, and proposed the skeleton*: K.Z. *Wrote the original manuscript*: Q.L., W.Z., and R.J. *Applied for copyright transfer*: Q.L., X.L., and Z.L. *Selected and organized the figures*: Q.L. and K.Z. *Revised the manuscript: K.Z*. All authors commented on this manuscript.

## References

[1] M. Latifi , A. Hay , J. Carroll , N. Dervisis , L. Arnold , S. L. Coutermarsh‐Ott , K. R. Kierski , S. Klahn , I. C. Allen , E. Vlaisavljevich , J. Tuohy , Vet. Comp. Oncol. 2021, 19, 411. 34057278 10.1111/vco.12742

[2] R. L. Siegel , K. D. Miller , H. E. Fuchs , A. Jemal , CA‐Cancer J. Clin. 2022, 72, 7. 35020204 10.3322/caac.21708

[3] R. Baskar , K. A. Lee , R. Yeo , K. W. Yeoh , Int. J. Med. Sci. 2012, 9, 193. 22408567 10.7150/ijms.3635 PMC3298009

[4] J. E. Krook , C. G. Moertel , L. L. Gunderson , H. S. Wieand , R. T. Collins , R. W. Beart , T. P. Kubista , M. A. Poon , W. C. Meyers , J. A. Mailliard , D. I. Twito , R. F. Morton , M. H. Veeder , T. E. Witzig , S. Cha , S. C. Vidyarthi , N. Engl. J. Med. 1991, 324, 709. 1997835 10.1056/NEJM199103143241101

[5] G. T. Wolf , N. Engl. J. Med. 1991, 324, 1685. 2034244 10.1056/NEJM199106133242402

[6] J. J. Shi , P. W. Kantoff , R. Wooster , O. C. Farokhzad , Nat. Rev. Cancer 2017, 17, 20. 27834398 10.1038/nrc.2016.108 PMC5575742

[7] C. L. Shapiro , A. Recht , N. Engl. J. Med. 2001, 344, 1997. 11430330 10.1056/NEJM200106283442607

[8] Y. Cao , C. Chen , Y. Tao , W. Lin , P. Wang , Pharmaceutics 2021, 13, 2003. 34959285 10.3390/pharmaceutics13122003 PMC8705248

[9] Z. F. Hu , Q. L. Wei , H. M. Zhang , W. N. Tang , Y. K. Kou , Y. Q. Sun , Z. C. Dai , X. W. Zheng , J. Mater. Chem. B 2022, 10, 339. 34951441 10.1039/d1tb02221k

[10] Y. Ding , Y. Wang , Q. Hu , Exploration 2022, 1, 20210106. 10.1002/EXP.20210106 PMC10190958 37323702

[11] L. P. Zhu , J. Liu , G. Y. Zhou , T. M. Liu , Y. L. Dai , G. J. Nie , Q. Zhao , Small 2021, 17, 2102624. 10.1002/smll.202102624 34378338

[12] Z. T. Li , Y. X. Wang , Y. X. Shen , C. G. Qian , D. Oupicky , M. J. Sun , Sci. Adv. 2020, 6, eaaz9240. 32440550 10.1126/sciadv.aaz9240 PMC7228744

[13] H. Wu , H. Li , Y. Liu , J. Liang , Q. Liu , Z. Xu , Z. Chen , X. Zhang , K. Zhang , C. Xu , Bioact. Mater. 2022, 13, 223. 35224304 10.1016/j.bioactmat.2021.10.048 PMC8843980

[14] G. Canti , A. Calastretti , A. Bevilacqua , E. Reddi , G. Palumbo , A. Nicolin , Neoplasma 2010, 57, 184. 20099984 10.4149/neo_2010_02_184

[15] Y. J. Ho , C. H. Wu , Q. F. Jin , C. Y. Lin , P. H. Chiang , N. Wu , C. H. Fan , C. M. Yang , C. K. Yeh , Biomaterials 2020, 232, 119723. 31891818 10.1016/j.biomaterials.2019.119723

[16] L. Lei , S. Cai , Y. Zhang , L. Yang , J. Deng , H. Mei , X. Zhang , K. Zhang , B. He , J. Cao , Adv. Funct. Mater. 2022, 32, 2103394.

[17] S. S. He , J. C. Li , P. H. Cheng , Z. L. Zeng , C. Zhang , H. W. Duan , K. Y. Pu , Angew. Chem., Int. Ed. 2021, 60, 19355. 10.1002/anie.202106392 34105217

[18] F. Kong , C. Fang , Y. Zhang , L. Duan , D. Du , G. Xu , X. Li , H. Li , Y. Yin , H. Xu , K. Zhang , Adv. Sci. 2022, 9, 2105523. 10.1002/advs.202105523 PMC8895135 35037431

[19] T. Y. Shang , X. Y. Yu , S. S. Han , B. Yang , Biomater. Sci. 2020, 8, 5241. 32996922 10.1039/d0bm01158d

[20] J. Zhang , M. Yang , X. Fan , M. Zhu , Y. Yin , H. Li , J. Chen , S. Qin , H. Zhang , K. Zhang , F. Yu , J. Nanobiotechnol. 2022, 20, 103. 10.1186/s12951-022-01324-w PMC8895626 35246159

[21] T. Wang , X. Xu , K. Zhang , Curr. Cancer Drug Targets 2021, 21, 545. 33618647 10.2174/1568009621666210219101552

[22] D. Jana , Y. Zhao , Exploration 2022, 2, 20210238. 10.1002/EXP.20210238 PMC10191001 37323881

[23] Y. Tao , E. G. Ju , Z. Liu , K. Dong , J. S. Ren , X. G. Qu , Biomaterials 2014, 35, 6646. 24818880 10.1016/j.biomaterials.2014.04.073

[24] X. X. Yao , B. C. Yang , S. Wang , Z. C. Dai , D. S. Zhang , X. W. Zheng , Q. Y. Liu , J. Mater. Chem. B 2020, 8, 8010. 32766612 10.1039/d0tb00411a

[25] H. Mei , X. Zhang , S. Cai , X. Zhang , Y. Zhang , Z. Guo , W. Shi , R. Chu , K. Zhang , J. Cao , B. He , Nano Today 2021, 41, 101305.

[26] Y. F. Zhang , Y. Y. Liao , Q. N. Tang , J. Lin , P. Huang , Angew. Chem., Int. Ed. 2021, 60, 10647. 10.1002/anie.202015590 33555085

[27] Y. C. Li , J. Xie , W. Um , D. G. You , S. Kwon , L. B. Zhang , J. T. Zhu , J. H. Park , Adv. Funct. Mater. 2021, 31, 2008061.

[28] Y. Yin , X. Jiang , L. Sun , H. Li , C. Su , Y. Zhang , G. Xu , X. Li , C. Zhao , Y. Chen , H. Xu , K. Zhang , Nano Today 2021, 36, 101009.

[29] M. Chen , H. Liao , Z. Bu , D. Wang , C. Fang , X. Liang , H. Li , J. Liu , K. Zhang , D. Su , Chem. Eng. J. 2022, 441, 136030.

[30] K. Zhang , H. R. Chen , X. S. Guo , D. Zhang , Y. Y. Zheng , H. R. Zheng , J. L. Shi , Sci. Rep. 2015, 5, 8766. 25739832 10.1038/srep08766 PMC4350106

[31] K. Zhang , Y. Cheng , W. W. Ren , L. P. Sun , C. Liu , D. Wang , L. H. Guo , H. X. Xu , Y. X. Zhao , Adv. Sci. 2018, 5, 10. 10.1002/advs.201800021 PMC6145269 30250780

[32] K. Zhang , H.‐Y. Li , J.‐Y. Lang , X.‐T. Li , W.‐W. Yue , Y.‐F. Yin , D. Du , Y. Fang , H. Wu , Y.‐X. Zhao , C. Xu , Adv. Funct. Mater. 2019, 29, 1905124.

[33] S. Rakoff‐Nahoum , R. Medzhitov , Nat. Rev. Cancer 2009, 9, 57. 19052556 10.1038/nrc2541

[34] Y. Wang , S. Zhang , H. Li , H. Wang , T. Zhang , M. R. Hutchinson , H. Yin , X. Wang , Acc. Chem. Res. 2020, 53, 1046. 32233400 10.1021/acs.accounts.9b00631

[35] M. K. Vidya , V. G. Kumar , V. Sejian , M. Bagath , G. Krishnan , R. Bhatta , Int. Rev. Immunol. 2018, 37, 20. 29028369 10.1080/08830185.2017.1380200

[36] A. Keshavarz , A. Pourbagheri‐Sigaroodi , P. Zafari , N. Bagheri , S. H. Ghaffari , D. Bashash , IUBMB Life 2021, 73, 10. 33217774 10.1002/iub.2412

[37] D. F. R. Boehmer , S. Formisano , C. C. de Oliveira Mann , S. A. Mueller , M. Kluge , P. Metzger , M. Rohlfs , C. Hörth , L. Kocheise , S. F. Lichtenthaler , K.‐P. Hopfner , S. Endres , S. Rothenfusser , C. C. Friedel , P. Duewell , M. Schnurr , L. M. Koenig , Sci. Immunol. 2021, 6, eabe2550. 34272227 10.1126/sciimmunol.abe2550

[38] D. Thoresen , W. Wang , D. Galls , R. Guo , L. Xu , A. M. Pyle , Immunol. Rev. 2021, 304, 154. 34514601 10.1111/imr.13022 PMC9293153

[39] B. R. Sharma , T. D. Kanneganti , Nat. Immunol. 2021, 22, 550. 33707781 10.1038/s41590-021-00886-5 PMC8132572

[40] S. S. Faria , S. Costantini , V. C. C. de Lima , V. P. de Andrade , M. Rialland , R. Cedric , A. Budillon , K. G. Magalhaes , J. Biomed. Sci. 2021, 28, 26. 33840390 10.1186/s12929-021-00724-8 PMC8040227

[41] R. Karki , S. M. Man , T. D. Kanneganti , Cancer Immunol. Res. 2017, 5, 94. 28093447 10.1158/2326-6066.CIR-16-0269 PMC5593081

[42] M. Ju , J. Bi , Q. Wei , L. Jiang , Q. Guan , M. Zhang , X. Song , T. Chen , J. Fan , X. Li , M. Wei , L. Zhao , Brief Bioinform 2021, 22, bbaa345. 33212483 10.1093/bib/bbaa345 PMC8294515

[43] J. Kwon , S. F. Bakhoum , Cancer Discovery 2020, 10, 26. 31852718 10.1158/2159-8290.CD-19-0761 PMC7151642

[44] A. Decout , J. D. Katz , S. Venkatraman , A. Ablasser , Nat. Rev. Immunol. 2021, 21, 548. 33833439 10.1038/s41577-021-00524-z PMC8029610

[45] Y. Chen , Q. Yin , X. F. Ji , S. J. Zhang , H. R. Chen , Y. Y. Zheng , Y. Sun , H. Y. Qu , Z. Wang , Y. P. Li , X. Wang , K. Zhang , L. L. Zhang , J. L. Shi , Biomaterials 2012, 33, 7126. 22789722 10.1016/j.biomaterials.2012.06.059

[46] Y. Wang , K. Zhang , Y. H. Xua , H. R. Chen , ACS Biomater. Sci. Eng. 2018, 4, 248. 33418692 10.1021/acsbiomaterials.7b00779

[47] K. Zhang , H. R. Chen , P. Li , X. W. Bo , X. L. Li , Z. Zeng , H. X. Xu , ACS Appl. Mater. Interfaces 2015, 7, 18590. 26245739 10.1021/acsami.5b04999

[48] S. Son , J. H. Kim , X. Wang , C. Zhang , S. A. Yoon , J. Shin , A. Sharma , M. H. Lee , L. Cheng , J. Wu , J. S. Kim , Chem. Soc. Rev. 2020, 49, 3244. 32337527 10.1039/c9cs00648f

[49] T. Inui , K. Makita , H. Miura , A. Matsuda , D. Kuchiike , K. Kubo , M. Mette , Y. Uto , T. Nishikata , H. Hori , N. Sakamoto , Anticancer Res. 2014, 34, 4589. 25075104

[50] T. Inui , H. Amitani , K. Kubo , D. Kuchiike , Y. Uto , T. Nishikata , M. Mette , Anticancer Res. 2016, 36, 3767. 27354652

[51] X. Guan , H.‐H. Yin , X.‐H. Xu , G. Xu , Y. Zhang , B.‐G. Zhou , W.‐W. Yue , C. Liu , L.‐P. Sun , H.‐X. Xu , K. Zhang , Adv. Funct. Mater. 2020, 30, 2000326.

[52] X. Lin , J. Song , X. Chen , H. Yang , Angew. Chem., Int. Ed. 2020, 59, 14212. 10.1002/anie.201906823 31267634

[53] I. Rosenthal , J. Z. Sostaric , P. Riesz , Ultrason. Sonochem. 2004, 11, 349. 15302020 10.1016/j.ultsonch.2004.03.004

[54] H. Shibaguchi , H. Tsuru , M. Kuroki , M. Kuroki , Anticancer Res. 2011, 31, 2425. 21873154

[55] J. E. Kennedy , Nat. Rev. Cancer 2005, 5, 321. 15776004 10.1038/nrc1591

[56] P. Zhao , Y. Deng , G. Xiang , Y. Liu , Int. J. Nanomed. 2021, 16, 4615. 10.2147/IJN.S307885 PMC8275046 34262272

[57] L. Duan , L. Yang , J. Jin , F. Yang , D. Liu , K. Hu , Q. X. Wang , Y. B. Yue , N. Gu , Theranostics 2020, 10, 462. 31903132 10.7150/thno.37593 PMC6929974

[58] L. Rengeng , Z. Qianyu , L. Yuehong , P. Zhongzhong , L. Libo , Photodiagn. Photodyn. Ther. 2017, 19, 159. 10.1016/j.pdpdt.2017.06.003 28606724

[59] Q. Wu , F. Zhang , X. Pan , Z. Huang , Z. Zeng , H. Wang , J. Jiao , X. Xiong , L. Bai , D. Zhou , H. Liu , Adv. Mater. 2021, 33, 2007073. 10.1002/adma.202007073 33987928

[60] X. Han , J. Chen , J. Chu , C. Liang , Q. Ma , Q. Fan , Z. Liu , C. Wang , J. Controlled Release 2019, 304, 233. 10.1016/j.jconrel.2019.05.008 31071371

[61] S. Yang , X. Wang , P. He , A. Xu , G. Wang , J. Duan , Y. Shi , G. Ding , Small 2021, 17, 2004867. 10.1002/smll.202004867 33511794

[62] X. Lin , R. Huang , Y. Huang , K. Wang , H. Li , Y. Bao , C. Wu , Y. Zhang , X. Tian , X. Wang , Int. J. Nanomed. 2021, 16, 1889. 10.2147/IJN.S290796 PMC7943542 33707944

[63] Q. Li , B. Lin , Y. Li , N. Lu , Int. J. Nanomed. 2021, 16, 3875. 10.2147/IJN.S301855 PMC8197575 34135582

[64] W. Chen , C. Liu , X. Ji , J. Joseph , Z. Tang , J. Ouyang , Y. Xiao , N. Kong , N. Joshi , O. C. Farokhzad , W. Tao , T. Xie , Angew. Chem., Int. Ed. 2021, 60, 7155. 10.1002/anie.202016330 33434327

[65] D. G. You , V. G. Deepagan , W. Um , S. Jeon , S. Son , H. Chang , H. I. Yoon , Y. W. Cho , M. Swierczewska , S. Lee , M. G. Pomper , I. C. Kwon , K. Kim , J. H. Park , Sci. Rep. 2016, 6, 23200. 26996446 10.1038/srep23200 PMC4800401

[66] Y. Zhu , W. Hong , X. Liu , L. Tan , J. Wu , C. Mao , Y. Xiang , S. Wu , K. M. C. Cheung , K. W. K. Yeung , Nanoscale 2021, 13, 15699. 34529746 10.1039/d1nr04512a

[67] X. Han , J. Huang , X. Jing , D. Yang , H. Lin , Z. Wang , P. Li , Y. Chen , ACS Nano 2018, 12, 4545. 29697960 10.1021/acsnano.8b00899

[68] W. Guan , L. Tan , X. Liu , Z. Cui , Y. Zheng , K. W. K. Yeung , D. Zheng , Y. Liang , Z. Li , S. Zhu , X. Wang , S. Wu , Adv. Mater. 2021, 33, 2006047. 10.1002/adma.202006047 33349987

[69] X. Wang , X. Wang , Q. Yue , H. Xu , X. Zhong , L. Sun , G. Li , Y. Gong , N. Yang , Z. Wang , Z. Liu , L. Cheng , Nano Today 2021, 39, 101170.

[70] S. Lu , W. Feng , C. Dong , X. Song , X. Gao , J. Guo , Y. Chen , Z. Hu , Adv. Healthcare Mater. 2022, 11, 2102135. 10.1002/adhm.202102135 34787379

[71] F. Gong , L. Cheng , N. Yang , Y. Gong , Y. Ni , S. Bai , X. Wang , M. Chen , Q. Chen , Z. Liu , Nat. Commun. 2020, 11, 3712. 32709842 10.1038/s41467-020-17485-x PMC7381661

[72] P. Zhu , Y. Chen , J. Shi , Adv. Mater. 2020, 32, 2001976. 10.1002/adma.202001976 32537778

[73] S. Liang , B. Liu , X. Xiao , M. Yuan , L. Yang , P. a. Ma , Z. Cheng , J. Lin , Adv. Mater. 2021, 33, 2101467. 10.1002/adma.202101467 34296464

[74] G. Li , X. Zhong , X. Wang , F. Gong , H. Lei , Y. Zhou , C. Li , Z. Xiao , G. Ren , L. Zhang , Z. Dong , Z. Liu , L. Cheng , Bioact. Mater. 2022, 8, 409. 34541410 10.1016/j.bioactmat.2021.06.021 PMC8429621

[75] Y. Wang , Y. Xu , S. Dong , P. Wang , W. Chen , Z. Lu , D. Ye , B. Pan , D. Wu , C. D. Vecitis , G. Gao , Nat. Commun. 2021, 12, 3508. 34108484 10.1038/s41467-021-23921-3 PMC8190189

[76] P. Wang , Q. Tang , L. Zhang , M. Xu , L. Sun , S. Sun , J. Zhang , S. Wang , X. Liang , ACS Nano 2021, 15, 11326. 10.1021/acsnano.1c00616 34180675

[77] K. Zhang , H. X. Xu , H. R. Chen , X. Q. Jia , S. G. Zheng , X. J. Cai , R. H. Wang , J. Mou , Y. Y. Zheng , J. L. Shi , Theranostics 2015, 5, 1291. 26379793 10.7150/thno.12691 PMC4568455

[78] J. Deprez , G. Lajoinie , Y. Engelen , S. C. De Smedt , I. Lentacker , Adv. Drug Delivery Rev. 2021, 172, 9. 10.1016/j.addr.2021.02.015 33705877

[79] Y. Zhang , Y. Yin , W. Zhang , H. Li , T. Wang , H. Yin , L. Sun , C. Su , K. Zhang , H. Xu , J. Nanobiotechnol. 2021, 19, 161. 10.1186/s12951-021-00897-2 PMC8166117 34059078

[80] T. Luo , D. Wang , L. Liu , Y. Zhang , C. Han , Y. Xie , Y. Liu , J. Liang , G. Qiu , H. Li , D. Su , J. Liu , K. Zhang , Adv. Sci. 2021, 8, 2101065. 10.1002/advs.202101065 PMC8498884 34369112

[81] J. Chen , H. L. Luo , Y. Liu , W. Zhang , H. X. Li , T. Luo , K. Zhang , Y. X. Zhao , J. J. Liu , ACS Nano 2017, 11, 12849. 29236476 10.1021/acsnano.7b08225

[82] Y. Pu , B. Zhou , H. Xiang , W. Wu , H. Yin , W. Yue , Y. Yin , H. Li , Y. Chen , H. Xu , Biomaterials 2020, 259, 120329. 32836058 10.1016/j.biomaterials.2020.120329

[83] H. Hu , W. Feng , X. Qian , L. Yu , Y. Chen , Y. Li , Adv. Mater. 2021, 33, 2005062. 10.1002/adma.202005062 33565157

[84] S. Wang , R. Tian , X. Zhang , G. Cheng , P. Yu , J. Chang , X. Chen , Adv. Mater. 2021, 33, 2007488. 10.1002/adma.202007488 33987898

[85] K. Zhang , H. X. Xu , X. Q. Jia , Y. Chen , M. Ma , L. P. Sun , H. R. Chen , ACS Nano 2016, 10, 10816. 28024356 10.1021/acsnano.6b04921

[86] Y. Pu , H. Yin , C. Dong , H. Xiang , W. Wu , B. Zhou , D. Du , Y. Chen , H. Xu , Adv. Mater. 2021, 33, 2104641. 10.1002/adma.202104641 34536041

[87] Y. Chen , H. Shang , C. Wang , J. Zeng , S. Zhang , B. Wu , W. Cheng , Int. J. Nanomed. 2022, 17, 105. 10.2147/IJN.S343361 PMC8752973 35027829

[88] Q. Xu , G. Zhan , Z. Zhang , T. Yong , X. Yang , L. Gan , Theranostics 2021, 11, 1937. 33408790 10.7150/thno.45511 PMC7778611

[89] Y. Tan , S. Yang , Y. Ma , J. Li , Q. Xie , C. Liu , Y. Zhao , Int. J. Nanomed. 2021, 16, 3241. 10.2147/IJN.S305857 PMC8121678 34007176

[90] F. Shen , D. Tao , R. Peng , Y. He , Z. Liu , J. Ji , L. Feng , Biomaterials 2022, 283, 121428. 35219148 10.1016/j.biomaterials.2022.121428

[91] C. Ji , J. Si , Y. Xu , W. Zhang , Y. Yang , X. He , H. Xu , X. Mou , H. Ren , H. Guo , Theranostics 2021, 11, 8587. 34373760 10.7150/thno.62572 PMC8344010

[92] Y. Si , J. Yue , Z. Liu , M. Li , F. Du , X. Wang , Z. Dai , N. Hu , J. Ju , S. Gao , X. Wang , P. Yuan , Int. J. Nanomed. 2021, 16, 1913. 10.2147/IJN.S297933 PMC7943766 33707946

[93] W. Xie , S. Zhu , B. Yang , C. Chen , S. Chen , Y. Liu , X. Nie , L. Hao , Z. Wang , J. Sun , S. Chang , Int. J. Nanomed. 2019, 14, 9377. 10.2147/IJN.S208404 PMC6896924 31819438

[94] H. Chen , L. Liu , A. Ma , T. Yin , Z. Chen , R. Liang , Y. Qiu , M. Zheng , L. Cai , Biomaterials 2021, 269, 120639. 33434714 10.1016/j.biomaterials.2020.120639

[95] Z. Zhang , B. Li , L. Xie , W. Sang , H. Tian , J. Li , G. Wang , Y. Dai , ACS Nano 2021, 15, 16934. 34661387 10.1021/acsnano.1c08026

[96] J. Wang , J. Huang , W. Zhou , J. Zhao , Q. Peng , L. Zhang , Z. Wang , P. Li , R. Li , J. Nanobiotechnol. 2021, 19, 87. 10.1186/s12951-021-00837-0 PMC7995598 33771168

[97] T. Zhang , Q. Zheng , Y. Fu , C. Xie , G. Fan , Y. Wang , Y. Wu , X. Cai , G. Han , X. Li , J. Nanobiotechnol. 2021, 19, 358. 10.1186/s12951-021-01105-x PMC8569996 34736483

[98] J. Zheng , Y. Sun , T. Long , D. Yuan , S. Yue , N. Zhang , Z. Yang , Drug Delivery 2022, 29, 1164. 35393920 10.1080/10717544.2022.2058653 PMC9004507

[99] B. Wu , Y. Yuan , J. Liu , H. Shang , J. Dong , X. Liang , D. Wang , Y. Chen , C. Wang , Y. Zhou , H. Jing , W. Cheng , J. Nanobiotechnol. 2021, 19, 177. 10.1186/s12951-021-00923-3 PMC8199394 34118951

[100] X. Qiao , L. Xue , H. Huang , X. Dai , Y. Chen , H. Ding , J. Nanobiotechnol. 2022, 20, 186. 10.1186/s12951-022-01398-6 PMC9003983 35413839

[101] M. Liu , A. R. Khan , J. Ji , G. Lin , X. Zhao , G. Zhai , J. Controlled Release 2018, 290, 150. 10.1016/j.jconrel.2018.10.007 30308258

[102] X. Duan , C. Chan , W. Lin , Angew. Chem., Int. Ed. 2019, 58, 670. 10.1002/anie.201804882 PMC7837455 30016571

[103] R. S. Riley , C. H. June , R. Langer , M. J. Mitchell , Nat. Rev. Drug Discovery 2019, 18, 175. 30622344 10.1038/s41573-018-0006-z PMC6410566

[104] H. Locy , S. de Mey , W. de Mey , M. De Ridder , K. Thielemans , S. K. Maenhout , Front. Immunol. 2018, 9, 2909. 30619273 10.3389/fimmu.2018.02909 PMC6297829

[105] X. Wen , C. Shi , X. Zeng , L. Zhao , L. Yao , Z. Liu , L. Feng , D. Zhang , J. Huang , Y. Li , Q. Lin , H. Chen , R. Zhuang , X. Chen , X. Zhang , Z. Guo , Clin. Cancer Res. 2022. 10.1158/1078-0432.CCR-22-0159 35320358

[106] X. Dai , Y. Gao , W. Wei , Semin. Cancer Biol. 2021, 10.1016/j.semcancer.2021.04.002. PMC8490479 33831533

[107] M. Yi , M. Niu , L. Xu , S. Luo , K. Wu , J. Hematol. Oncol. 2021, 14, 10. 33413496 10.1186/s13045-020-01027-5 PMC7792099

[108] R. Grosser , L. Cherkassky , N. Chintala , P. S. Adusumilli , Cancer Cell 2019, 36, 471. 31715131 10.1016/j.ccell.2019.09.006 PMC7171534

[109] L. M. Colli , M. J. Machiela , H. Zhang , T. A. Myers , L. Jessop , O. Delattre , K. Yu , S. J. Chanock , Cancer Res. 2017, 77, 3666. 28446466 10.1158/0008-5472.CAN-16-3338 PMC5522610

[110] J. Galon , D. Bruni , Nat. Rev. Drug Discovery 2019, 18, 197. 30610226 10.1038/s41573-018-0007-y

[111] W. W. Yue , L. Chen , L. D. Yu , B. G. Zhou , H. H. Yin , W. W. Ren , C. Liu , L. H. Guo , Y. F. Zhang , L. P. Sun , K. Zhang , H. X. Xu , Y. Chen , Nat. Commun. 2019, 10, 2025. 31048681 10.1038/s41467-019-09760-3 PMC6497709

[112] X. Li , S. Khorsandi , Y. Wang , J. Santelli , K. Huntoon , N. Nguyen , M. Yang , D. Lee , Y. Lu , R. Gao , B. Y. S. Kim , C. de Gracia Lux , R. F. Mattrey , W. Jiang , J. Lux , Nat. Nanotechnol. 2022, 17, 891. 35637356 10.1038/s41565-022-01134-z PMC9378430

[113] H. Zhang , X. Pan , Q. Wu , J. Guo , C. Wang , H. Liu , Exploration 2021, 1, 20210010. 10.1002/EXP.20210010 PMC10190974 37323218

[114] C. Li , Y. Lu , L. Cheng , X. Zhang , J. Yue , J. Liu , Mol. Pharmaceutics 2021, 18, 2091. 10.1021/acs.molpharmaceut.1c00229 33886331

[115] S. Dababou , C. Marrocchio , R. Scipione , H. P. Erasmus , P. Ghanouni , M. Anzidei , C. Catalano , A. Napoli , Radiographics 2018, 38, 603. 29394144 10.1148/rg.2018170129

[116] G. Mauri , L. Nicosia , Z. Xu , S. Di Pietro , L. Monfardini , G. Bonomo , G. M. Varano , F. Prada , P. D. Vigna , F. Orsi , Br. J. Radiol. 2018, 91, 20170641. 29168922 10.1259/bjr.20170641 PMC5965486

[117] M. Marinova , S. Ghaei , F. Recker , T. Tonguc , O. Kaverina , O. Savchenko , D. Kravchenko , M. Thudium , C. C. Pieper , E. K. Egger , A. Mustea , U. Attenberger , R. Conrad , D. R. Hadizadeh , H. Strunk , Int. J. Hyperthermia 2021, 38, 30. 10.1080/02656736.2021.1939444 34420447

[118] T. Deng , L. Zhang , X. Li , J. I. Zink , H. H. Wu , ACS Nano 2021, 15, 14618. 34519214 10.1021/acsnano.1c04339

[119] X. Zhang , L. Landgraf , N. Bailis , M. Unger , T. H. Jochimsen , A. Melzer , J. Nucl. Med. 2021, 62, 1181. 34088775 10.2967/jnumed.120.256230 PMC8882895

[120] S. Abe , H. Nagata , E. J. Crosby , Y. Inoue , K. Kaneko , C. X. Liu , X. Yang , T. Wang , C. R. Acharya , P. Agarwal , J. Snyder , W. Gwin , M. A. Morse , P. Zhong , H. K. Lyerly , T. Osada , J. Immunother. Cancer 2022, 10, e003717. 35039461 10.1136/jitc-2021-003717 PMC8765068

[121] G. Shi , M. Zhong , F. Ye , X. Zhang , Cancer Biol. Med. 2019, 16, 714. 31908890 10.20892/j.issn.2095-3941.2019.0232 PMC6936245

[122] L. Huang , Y. Li , Y. Du , Y. Zhang , X. Wang , Y. Ding , X. Yang , F. Meng , J. Tu , L. Luo , C. Sun , Nat. Commun. 2019, 10, 4871. 31653838 10.1038/s41467-019-12771-9 PMC6814770

[123] T. Tonguc , H. Strunk , M. A. Gonzalez‐Carmona , F. Recker , D. Lutjohann , M. Thudium , R. Conrad , M. U. Becher , O. Savchenko , D. Davidova , G. Luechters , A. Mustea , C. P. Strassburg , U. Attenberger , C. C. Pieper , J. Jenne , M. Marinova , Int. J. Hyperthermia 2021, 38, 65. 10.1080/02656736.2021.1900926 34420445

[124] X. Q. Zhu , P. Lu , Z. L. Xu , Q. Zhou , J. Zhang , Z. B. Wang , F. Wu , Cells 2021, 10, 3346. 34943854 10.3390/cells10123346 PMC8699337

[125] A. Eranki , P. Srinivasan , M. Ries , A. Kim , C. A. Lazarski , C. T. Rossi , T. D. Khokhlova , E. Wilson , S. M. Knoblach , K. V. Sharma , B. J. Wood , C. Moonen , A. D. Sandler , P. C. W. Kim , Clin. Cancer Res. 2020, 26, 1152. 31615935 10.1158/1078-0432.CCR-19-1604 PMC9009723

[126] P. X. E. Mouratidis , M. Costa , I. Rivens , E. E. Repasky , G. T. Haar , J. R. Soc., Interface 2021, 18, 20210266. 34229458 10.1098/rsif.2021.0266 PMC8261215

[127] V. S. Bachu , J. Kedda , I. Suk , J. J. Green , B. Tyler , Ann. Biomed. Eng. 2021, 49, 1975. 34374945 10.1007/s10439-021-02833-9 PMC8608284

[128] N. D. Sheybani , A. R. Witter , E. A. Thim , H. Yagita , T. N. J. Bullock , R. J. Price , J. Immunother. Cancer 2020, 8, e001008. 32819975 10.1136/jitc-2020-001008 PMC7443308

[129] D. Wang , F. Jiang , L. Wang , Y. Tang , Z. Zhang , Y. Du , J. Zou , Biochem. Biophys. Res. Commun. 2021, 571, 104. 34314995 10.1016/j.bbrc.2021.07.061

[130] J. Shen , J. Hao , Y. Chen , H. Liu , J. Wu , B. Hu , Y. Wang , Y. Zheng , X. Cai , J. Nanobiotechnol. 2021, 19, 345. 10.1186/s12951-021-01087-w PMC8555249 34715854

[131] K. J. Pahk , C. H. Shin , I. Y. Bae , Y. Yang , S. H. Kim , K. Pahk , H. Kim , S. J. Oh , Sci. Rep. 2019, 9, 9050. 31227775 10.1038/s41598-019-45542-z PMC6588624

[132] M. Ma , H. X. Xu , H. R. Chen , X. Q. Jia , K. Zhang , Q. Wang , S. G. Zheng , R. Wu , M. H. Yao , X. J. Cai , F. Q. Li , J. L. Shi , Adv. Mater. 2014, 26, 7378. 25228225 10.1002/adma.201402969

[133] X. Wang , H. R. Chen , Y. Chen , M. Ma , K. Zhang , F. Q. Li , Y. Y. Zheng , D. P. Zeng , Q. Wang , J. L. Shi , Adv. Mater. 2012, 24, 785. 22223403 10.1002/adma.201104033

[134] K. Zhang , H. R. Chen , F. Q. Li , Q. Wang , S. G. Zheng , H. X. Xu , M. Ma , X. Q. Jia , Y. Chen , J. Mou , X. Wang , J. L. Shi , Biomaterials 2014, 35, 5875. 24746229 10.1016/j.biomaterials.2014.03.043

[135] X. Wang , H. R. Chen , Y. Y. Zheng , M. Ma , Y. Chen , K. Zhang , D. P. Zeng , J. L. Shi , Biomaterials 2013, 34, 2057. 23246067 10.1016/j.biomaterials.2012.11.044

[136] M. P. Singh , S. N. Sethuraman , C. Miller , J. Malayer , A. Ranjan , Theranostics 2021, 11, 540. 33391491 10.7150/thno.49517 PMC7738858

[137] S. Qu , T. Worlikar , A. E. Felsted , A. Ganguly , M. V. Beems , R. Hubbard , A. L. Pepple , A. A. Kevelin , H. Garavaglia , J. Dib , M. Toma , H. Huang , A. Tsung , Z. Xu , C. S. Cho , J. Immunother. Cancer 2020, 8, e000200. 31940590 10.1136/jitc-2019-000200 PMC7057529

[138] X. Kuai , Y. Zhu , Z. Yuan , S. Wang , L. Lin , X. Ye , Y. Lu , Y. Luo , Z. Pang , D. Geng , B. Yin , Acta Pharm. Sin. B 2022, 12, 967. 35256958 10.1016/j.apsb.2021.07.025 PMC8897201

[139] X. Duan , M. Wang , X. Han , J. Ren , G. Huang , S. Ju , Q. Zhang , Cell Cycle 2020, 19, 3595. 33283623 10.1080/15384101.2020.1853942 PMC7781627

[140] Y. Shen , L. Chen , X. Guan , X. Han , X. Bo , S. Li , L. Sun , Y. Chen , W. Yue , H. Xu , ACS Nano 2021, 15, 20414. 34881574 10.1021/acsnano.1c08826

[141] S. Huang , T. Li , Y. Chen , J. Liu , Y. Wang , C. Yang , C. Wang , S. Ju , Y. Bai , W. Yao , B. Xiong , Int. J. Hyperthermia 2022, 39, 278. 35129044 10.1080/02656736.2022.2032406

[142] H. Xu , W. Sun , Y. Kong , Y. Huang , Z. Wei , L. Zhang , J. Liang , X. Ye , J. Cancer Res. Ther. 2020, 16, 1718. 33565523 10.4103/jcrt.JCRT_1399_20

[143] Z. Wei , X. Yang , X. Ye , J. Cancer Res. Ther. 2020, 16, 1191. 33004770 10.4103/jcrt.JCRT_798_20

[144] M. Yu , H. Pan , N. Che , L. Li , C. Wang , Y. Wang , G. Ma , M. Qian , J. Liu , M. Zheng , H. Xie , L. Ling , Y. Zhao , X. Guan , Q. Ding , W. Zhou , S. Wang , Cell Mol. Immunol. 2021, 18, 2153. 32385362 10.1038/s41423-020-0449-0 PMC8429677

[145] H. Takahashi , E. Berber , H. Surg , Surg. Nutr. 2020, 9, 49. 10.21037/hbsn.2019.06.08 PMC7026789 32140478

[146] F. E. F. Timmer , B. Geboers , S. Nieuwenhuizen , E. A. C. Schouten , M. Dijkstra , J. J. J. de Vries , M. P. van den Tol , T. D. de Gruijl , H. J. Scheffer , M. R. Meijerink , Curr. Oncol. Rep. 2021, 23, 68. 33864144 10.1007/s11912-021-01057-3 PMC8052234

[147] W. Zhou , M. Yu , H. Pan , W. Qiu , H. Wang , M. Qian , N. Che , K. Zhang , X. Mao , L. Li , R. Wang , H. Xie , L. Ling , Y. Zhao , X. Liu , C. Wang , Q. Ding , S. Wang , J. Immunother. Cancer 2021, 9, e002343. 33795388 10.1136/jitc-2021-002343 PMC8021888

[148] L. Dumolard , J. Ghelfi , G. Roth , T. Decaens , Z. M. Jilkova , Int. J. Mol. Sci. 2020, 21, 4398. 10.3390/ijms21124398 PMC7352237 32575734

[149] W. Zhou , M. Yu , X. Mao , H. Pan , X. Tang , J. Wang , N. Che , H. Xie , L. Ling , Y. Zhao , X. Liu , C. Wang , K. Zhang , W. Qiu , Q. Ding , S. Wang , Adv. Sci. 2022, 9, 2200033. 10.1002/advs.202200033 PMC9189675 35403824

[150] K. Leuchte , E. Staib , M. Thelen , P. Godel , A. Lechner , P. Zentis , M. Garcia‐Marquez , D. Waldschmidt , R. R. Datta , R. Wahba , C. Wybranski , T. Zander , A. Quaas , U. Drebber , D. L. Stippel , C. Bruns , M. von Bergwelt‐Baildon , K. Wennhold , H. A. Schlosser , Cancer Immunol. Immunother. 2021, 70, 893. 33006650 10.1007/s00262-020-02734-1 PMC7979675

[151] Y. Chen , H. Huang , Y. Li , W. Xiao , Y. Liu , R. Chen , Y. Zhu , X. Zheng , C. Wu , L. Chen , Front. Immunol. 2022, 13, 832230. 35320940 10.3389/fimmu.2022.832230 PMC8935077

[152] T. M. Wah , J. Zhong , M. Wilson , N. S. Vasudev , R. E. Banks , Cancers 2021, 13, 6037. 34885149 10.3390/cancers13236037 PMC8656737

[153] S. P. Wang , L. F. Tan , P. Liang , T. L. Liu , J. Z. Wang , C. H. Fu , J. Yu , J. P. Dou , L. Hong , X. W. Meng , J. Mater. Chem. B 2016, 4, 2133. 32263180 10.1039/c6tb00296j

[154] C. H. Fu , H. Q. Zhou , L. F. Tan , Z. B. Huang , Q. Wu , X. L. Ren , J. Ren , X. W. Meng , ACS Nano 2018, 12, 2201. 29286623 10.1021/acsnano.7b08868

[155] S. S. Tang , H. Q. Zhou , Q. Wu , C. H. Fu , L. F. Tan , X. L. Ren , Z. B. Huang , X. D. Chen , J. Ren , X. W. Meng , J. Mater. Chem. B 2017, 5, 9025. 32264130 10.1039/c7tb01472d

[156] Q. Hou , K. Zhang , S. Chen , J. Chen , Y. Zhang , N. Gong , W. Guo , C. Fang , L. Wang , J. Jiang , J. Dou , X. Liang , J. Yu , P. Liang , ACS Nano 2022, 16, 5704. 10.1021/acsnano.1c10714 35352557

[157] Q. Zhou , N. Gong , D. Zhang , J. Li , X. Han , J. Dou , J. Huang , K. Zhu , P. Liang , X. J. Liang , J. Yu , ACS Nano 2021, 15, 2920. 33523631 10.1021/acsnano.0c09120

[158] K. Wang , C. Wang , H. Jiang , Y. Zhang , W. Lin , J. Mo , C. Jin , Front. Immunol. 2021, 12, 792781. 34975896 10.3389/fimmu.2021.792781 PMC8714655

[159] C. Shao , M. Yang , Y. Pan , D. Xie , B. Chen , S. Ren , C. Zhou , Front. Immunol. 2021, 12, 696749. 34413851 10.3389/fimmu.2021.696749 PMC8368438

[160] H. Cao , L. Duan , Y. Zhang , J. Cao , K. Zhang , Signal Transduction Targeted Ther. 2021, 6, 426. 10.1038/s41392-021-00830-x PMC8674418 34916490

[161] L. Zheng , S. Liu , X. Cheng , Z. Qin , Z. Lu , K. Zhang , J. Zhao , Adv. Sci. 2019, 6, 1900099. 10.1002/advs.201900099 PMC6702628 31453055

[162] M. Yang , Y. Zhang , C. Fang , L. Song , Y. Wang , L. Lu , R. Yang , Z. Bu , X. Liang , K. Zhang , Q. Fu , Adv. Mater. 2022, 34, 2109522. 10.1002/adma.202109522 35120266

[163] K. Zhang , Y. Fang , Y. He , H. Yin , X. Guan , Y. Pu , B. Zhou , W. Yue , W. Ren , D. Du , H. Li , C. Liu , L. Sun , Y. Chen , H. Xu , Nat. Commun. 2019, 10, 5380. 31772164 10.1038/s41467-019-13115-3 PMC6879564

[164] Z. Yang , Y. Zhu , Z. Dong , W. Li , N. Yang , X. Wang , L. Feng , Z. Liu , Nat. Commun. 2021, 12, 4299. 34262038 10.1038/s41467-021-24604-9 PMC8280226

[165] P. Biondetti , L. Saggiante , A. M. Ierardi , M. Iavarone , A. Sangiovanni , F. Pesapane , E. M. Fumarola , P. Lampertico , G. Carrafiello , Cancers 2021, 13, 5797. 34830949 10.3390/cancers13225797 PMC8616392

[166] A. C. da Costa , M. Sodergren , K. Jayant , F. Santa Cruz , D. Spalding , M. Pai , N. Habib , World J. Gastroenterol. 2020, 26, 2040. 32536773 10.3748/wjg.v26.i17.2040 PMC7267689

[167] J. M. Llovet , T. De Baere , L. Kulik , P. K. Haber , T. F. Greten , T. Meyer , R. Lencioni , Nat. Rev. Gastroenterol. Hepatol. 2021, 18, 293. 33510460 10.1038/s41575-020-00395-0

[168] Y. Minami , N. Nishida , M. Kudo , Eur. Radiol. 2019, 29, 5045. 30963271 10.1007/s00330-019-06189-6

[169] E. G. Nesbit , E. D. Donnelly , J. B. Strauss , Curr. Treat. Options Oncol. 2021, 22, 94. 34426881 10.1007/s11864-021-00889-2

[170] H. Ogawa , T. Yajima , M. Sohda , K. Shirabe , H. Saeki , Ann. Gastroenterol. Surg. 2021, 5, 747. 34755006 10.1002/ags3.12472 PMC8560592

[171] X.‐W. Bo , F. Lu , H.‐X. Xu , L.‐P. Sun , K. Zhang , Front. Oncol. 2020, 10, 580431. 33194708 10.3389/fonc.2020.580431 PMC7658440

[172] L. R. Shi , J. J. Wang , N. H. Ding , Y. Zhang , Y. B. Zhu , S. L. Dong , X. H. Wang , C. L. Peng , C. H. Zhou , L. D. Zhou , X. D. Li , H. B. Shi , W. Wu , X. Y. Long , C. P. Wu , W. H. Liao , Nat. Commun. 2019, 10, 5421. 31780645 10.1038/s41467-019-13204-3 PMC6883042

[173] K. Zhang , P. Li , H. R. Chen , X. W. Bo , X. L. Li , H. X. Xu , ACS Nano 2016, 10, 2549. 26800221 10.1021/acsnano.5b07486

[174] K. Zhang , P. Li , Y. P. He , X. W. Bo , X. L. Li , D. D. Li , H. R. Chen , H. X. Xu , Biomaterials 2016, 99, 34. 27209261 10.1016/j.biomaterials.2016.05.014

[175] Y. Fang , H.‐Y. Li , H.‐H. Yin , S.‐H. Xu , W.‐W. Ren , S.‐S. Ding , W.‐Z. Tang , L.‐H. Xiang , R. Wu , X. Guan , K. Zhang , ACS Appl. Mater. Interfaces 2019, 11, 11251. 30874421 10.1021/acsami.9b02401

[176] C. Zang , Y. Zhao , L. Qin , G. Liu , J. Sun , K. Li , Y. Zhao , S. Sheng , H. Zhang , N. He , P. Zhao , Q. Wang , X. Li , Y. Peng , T. Dong , Y. Zhang , BMC Cancer 2021, 21, 1007. 34496797 10.1186/s12885-021-08720-9 PMC8428121

[177] C. Kole , N. Charalampakis , S. Tsakatikas , M. Vailas , D. Moris , E. Gkotsis , S. Kykalos , M. V. Karamouzis , D. Schizas , Cancers 2020, 12, 2859. 10.3390/cancers12102859 PMC7600093 33020428

[178] J. S. Yoon , B. G. Song , J. H. Lee , H. Y. Lee , S. W. Kim , Y. Chang , Y. B. Lee , E. J. Cho , S. J. Yu , D. H. Sinn , Y. J. Kim , J. H. Lee , J. H. Yoon , BMC Cancer 2019, 19, 523. 31151419 10.1186/s12885-019-5740-z PMC6543598

[179] R. Xing , J. Gao , Q. Cui , Q. Wang , Front. Immunol. 2021, 12, 783236. 34899747 10.3389/fimmu.2021.783236 PMC8660685

[180] M. Chen , Y. Tan , J. Hu , Y. Jiang , Z. Wang , Z. Liu , Q. Chen , Small 2021, 17, 2104773. 10.1002/smll.202104773 34729889

[181] J. W. Han , S. K. Yoon , Pharmaceutics 2021, 13, 1387.34575463

[182] M. H. Sodergren , N. Mangal , H. Wasan , A. Sadanandam , V. P. Balachandran , L. R. Jiao , N. Habib , J. Cancer Res. Clin. Oncol. 2020, 146, 2897. 32748119 10.1007/s00432-020-03332-5 PMC7519893

[183] K. W. Huang , C. P. Tan , V. Reebye , C. E. Chee , D. Zacharoulis , R. Habib , D. C. Blakey , J. J. Rossi , N. Habib , M. H. Sodergren , Int. J. Mol. Sci. 2021, 22, 9168. 34502076 10.3390/ijms22179168 PMC8431011

[184] P. Zeng , D. Shen , C. H. Zeng , X. F. Chang , G. J. Teng , Curr. Oncol. Rep. 2020, 22, 76. 32596779 10.1007/s11912-020-00943-6

[185] Y. Shi , T. Lammers , Acc. Chem. Res. 2019, 52, 1543. 31120725 10.1021/acs.accounts.9b00148 PMC7115879

[186] L. Racca , V. Cauda , Nano‐Micro Lett. 2020, 13, 11. 10.1007/s40820-020-00537-8 PMC8187688 34138198

